# Detection of Virulence-Associated Genes and *in vitro* Gene Transfer From *Aeromonas* sp. Isolated From Aquatic Environments of Sub-himalayan West Bengal

**DOI:** 10.3389/fvets.2022.887174

**Published:** 2022-06-10

**Authors:** Preeti Mangar, Partha Barman, Anoop Kumar, Aniruddha Saha, Dipanwita Saha

**Affiliations:** ^1^Department of Botany, University of North Bengal, Siliguri, India; ^2^Department of Biotechnology, University of North Bengal, Siliguri, India

**Keywords:** *Aeromonas*, 16S rRNA gene, virulence related genes, *Anabas testudineus*, cytotoxicity, conjugational efficiency, plasmid

## Abstract

*Aeromonas* is omnipresent in aquatic environments and cause disease within a wide host range. A total of thirty-four isolates from water samples of small fish farms were identified as *Aeromonas* based on biochemical characteristics and 16S rRNA gene sequence. A total of six virulent factors were analyzed which indicated 100% of isolates as beta-haemolytic and proteolytic, whereas 44.1, 38.2, and 70.6% of isolates produced DNAse, siderophore, and amylase, respectively. Studies on the occurrence of four genetic determinants of virulence factors revealed that *aer/haem* (haemolytic toxin) and *flaA* (polar flagella) genes were present in 44.1% of strains whereas *ascV* (type 3 secretion system) and *aspA* (serine protease) genes were detected in 21.5 and 8.82% of strains, respectively. Fish (*Anabas testudineus*) challenge studies showed that the isolate GP3 (*Aeromonas veronii*) bearing five virulent factors with the combination of *aer/haem*^+^*/ascV*^+^*/fla*^+^ genes induced severe lesions leading to 100% of mortality. In contrast, RB7 possessing four virulence factors and three genes (*aer/haem*^+^*/ascV*^+^*/aspA*^+^) could not produce severe lesions and any mortality indicating the absence of correlation between the virulence factors, its genes, and the pathogenicity in fishes. GP3 was cytotoxic to human liver cell line (WRL-68) in trypan blue dye exclusion assay. The 431 bp *aer/haem* gene of GP3 was transferable to *E. coli* Dh5α with a conjugational efficiency of 0.394 × 10^**–**4^ transconjugants per recipient cell. The transfer was confirmed by PCR and by the presence of 23-kb plasmids in both donor and transconjugants. Therefore, the occurrence of mobile genetic elements bearing virulence-associated genes in *Aeromonas* indicates the need for periodic monitoring of the aquatic habitat to prevent disease outbreaks.

## Introduction

The genus *Aeromonas* is a pervasive group of microorganisms in the aquatic environment (1). It has the ability to induce diseases in fishes, mammals, reptiles, and amphibians (2). *Aeromonas* has been recognized as an etiological agent of several pathogenic conditions such as motile aeromonad syndrome (MAS), haemorrhagic septicaemia, fin rot, red sore disease, and scale protrusions in fish (3). Additionally, its role as an opportunistic pathogen in immunocompromised human and in infections such as septicaemia, bacterial endocarditis, gastroenteritis, wound sepsis, and traveller's diarrhea cannot be undermined (4). At present, *Aeromonas* is an important water-borne pathogen and has gained significance with its ability to induce multiple infections in a wide host range (5). A total of 75% of freshwater fish production in India is contributed by the state of West Bengal (6). About 251 species of freshwater fishes are found here in various freshwater resources such as tanks, ponds, creeks, beels, barrages, reservoirs, small river streams, major rivers, and an interlinked river drainage system (7). However, the yield is largely affected by mortality due to infections. Several species of highly pathogenic motile aeromonads including *Aeromonas hydrophila, Aeromonas caviae*, and *Aeromonas sobria* were found to be associated with diseases with mass mortalities in fishes (8). Pathogenicity in *Aeromonas* spp. has been reported to be due to multiple virulence factors that contribute to different mechanisms of the infection process. These factors include haemolysins, proteases, elastases, lipases, enterotoxins, phospholipases, chitinases, and deoxyribonucleases which can cause tissue damage and lethality (9). The genes encoding virulence factors determine the pathogenicity of a microorganism (10, 11). Pore-forming toxin genes *aerA* (encoding aerolysin), *act* (encoding cytotoxic toxins), and *alt* and *ast* (encoding cytotonic enterotoxins) are the primary elements of virulence within *Aeromonas* spp. (12). In addition, virulence genes encoding serine protease (*ser, aspA*), polar flagella (*flaA*), lateral flagella (*lafA*), DNase (*exu*), elastase (*ahyB*), glycerophospholipid: cholesterol acyltransferase (*gCAT*), and type III secretion system (*ascV*) have also been traced within *Aeromonas* (13). The invasiveness and adherence of *Aeromonas hydrophila* to HEpG2 cell monolayers, Vero cells, Chinese hamster ovarian cells, and erythrocytes have also been documented (14). Mobile genetic elements that carry virulence factors have been reported as widespread in the aquatic environment among different genera of bacteria such as *Vibrio* (15, 16), *Photobacterium damselae* (17), and *Staphylococcus aureus* (18). Some pieces of evidence show that *Aeromonas* can actively participate in processes of transfer of genetic material (*via* conjugation) with phylogenetically distant bacteria (19). In a study on the whole-genome sequence, Poole et al. (20) observed that *A. hydrophila* may be capable of transferring resistance and virulence genes to other related genera in the environment. This study aims at the isolation of *Aeromonas* spp. from fish farming environments of North Bengal, India for investigating the occurrence and distribution of molecular markers responsible for virulence. The current work provides experimental evidence of the conjugational spread of virulence genes to *E. coli* Dh5α.

## Materials and Methods

### Sample Collection and Isolation of *Aeromonas* sp.

The *Aeromonas* populations from water samples of 9 different fish farming sites across Darjeeling and Jalpaiguri districts of Sub-Himalayan West Bengal were investigated. Samples were collected in sterilized vials, kept at 4°C, and analyzed within 24 h of procuring. For isolation of the bacteria, 0.1 ml of ten-fold diluted samples prepared in sterile distilled water was spread plated on *Aeromonas* isolation medium (HiMedia Laboratories Ltd., Mumbai, India). The plates were incubated for 24 h at 30°C in the bacterial incubator. Altogether, 83 single colonies were selected based on the expected morphology from the spread plates, and pure cultures were maintained by streaking them on tryptone soya agar slants (21). Glycerol stocks of cultures were maintained in 10% glycerol at −20°C. All of the isolates were initially screened presumptively using a slightly modified key test called Aerokey II, described by Carnahan et al. (22), to identify the genus *Aeromonas*. The isolates were tested for Gram-staining, oxidase, catalase, glucose fermentation, resistance to 0/129 discs, and esculin hydrolysis (23). The strains were labeled as presumptive *Aeromonas* spp. depending on the tests.

### Detection of Phenotypic Attributes of Virulence

Haemolytic activity of *Aeromonas* positive bacterial isolates was assessed by cultivating on tryptone soya agar (TSA) medium supplemented with 5% sheep blood (vol/vol) for 24 h at 30°C. Expression of haemolysin was depicted by a zone of clearance around bacterial colonies (24). Protease production was determined by the growth of bacterial isolates in nutrient agar containing 1.5% of skimmed milk and incubated for 24 h at 30°C. Casein-degrading bacteria showed clear zone around the colonies (24). DNase production was tested by inoculating the isolates in DNAse agar medium (HiMedia Laboratories, Mumbai) for 24 h at 30°C. The appearance of pale pink to white halos around the colonies indicated a positive result (25). Lipolytic activity was tested by growing the isolates in Tween-80 medium for 24 h at 30°C. Opaque zones around streaked cultures indicating crystal formation were considered positive for lipase (26). Amylase production was determined in starch agar plates following the method given by Barrow and Feltham (25). Siderophore production was determined using the Universal Chromazurol S (CAS) assay (27).

### Molecular Identification of the Isolates

#### Sequence Similarity and Phylogenetic Analysis

The bacterial isolates were identified by 16S rRNA gene sequencing using universal primers. The total genomic DNAs were isolated from all the strains by CTAB method (28). Amplification of the 16S rRNA gene was performed in 25 μl reaction using the universal primers 27F (5′-AGAGTTTGATCCTGGCTCAG-3′) and 1498R (5′-GGTTCACTTGTTACGACTT-3′) (29) following specific cycling conditions: initial denaturation at 95°C for 3 min, 30 cycles of denaturation at 95°C for 30 s, annealing at the temperature of 52.2°C for 30 s, extension at 72°C for 30 s, and a final extension at 72°C for 5 min on a thermal cycler (Applied Biosystems GeneAmp PCR 2400). The amplified product was purified using PCR purification kit (BR Biochem Life Sciences Pvt. Ltd) and sequenced at Eurofins sequencing services, Bangalore. All the obtained sequences were submitted to NCBI and GenBank accession numbers are given in the tree. The phylogenetic tree was constructed with the neighbor-joining method using MEGA 6.0 (30). Confidence in the tree topology was determined by bootstrap analysis using 1,000 resamplings of the sequences (31).

#### Pathogenicity Testing in Fishes

Healthy small-sized fishes (weight ~25–30 g) of *Anabas testudineus* were collected from fish farms in nearby areas of Sonapur and Gangarampur of Darjeeling district and maintained for 15 days in glass aquaria measuring 90 cm × 35 cm × 35 cm (10–12 fishes in each aquarium) for acclimatization. Water temperature was maintained at 25–30°C. For the fish pathogenicity testing, six strains (GP3, RB7, BP3, RJB1, MG8, and PP21) bearing two or more virulence properties and isolated from different locations were selected. The selected isolates were cultured in tryptic soy broth (HiMedia Laboratories, Mumbai) for 18 h at 30°C with constant shaking at 90 rpm. The cells were harvested as pellets following the centrifugation at 10,000 g for 10 min at 4°C and resuspended in 0.85% saline solution. The suspension was then adjusted to cell density of 1 × 10^7^cfu/ml by measuring O.D. at 600 nm (O.D. = 0.8) in a spectrophotometer. Prior to infection, the fishes were anesthetized by keeping in benzocaine solution (25 mg L^−1^) for 1–2 min. Intramuscular injection was given at 0.4 ml per 25 g of body weight with each of the prepared bacterial cell suspensions. The fishes injected with individual bacterium were maintained in separate aquaria (10 fishes in each aquarium). The control set of fishes kept in a separate aquarium was administered with 0.85% saline solution at similar dose. A negative control set was additionally maintained under similar conditions in which the fishes did not receive any injection. Development of ulcers and damage to the surface tissue were monitored every 24 h postadministration for 1 week (32). The Kaplan–Meier survival analysis was performed using the GraphPad Prism software (version 9.3.1).

#### Cytotoxicity Test

Cytotoxicity of the *Aeromonas* isolate GP3 was tested in WRL-68 cell lines. GP3 was grown in LB broth at 30°C for 16 h under shaking at 90 rpm. The resultant culture was centrifuged at 10,000 g for 30 min at 4°C and the cell-free supernatant was collected carefully and filtered through a 0.45-mm filter. Cell-free filtrates of a non-pathogenic strain *Lactobacillus* sp. were similarly prepared and included in the experiment as the positive control, whereas sterile LB medium was used for negative control. WRL-68 cell line (human, liver, embryonic) obtained from National Centre for Cell Science (NCCS) Pune, Maharashtra, India was grown in Dulbeco's modified Eagle's medium (DMEM) with 10% foetal calf serum in an atmosphere containing 5% CO_2_. For morphological examination, cells were seeded in 60-mm polyvinyl-coated culture plates and incubated at 37°C for 24 h in CO_2_ incubator. Next day, 1.5 ml of the two cell-free filtrates and sterile LB medium was added to the cells taken in three separate sets and incubated for 1 h at 25°C. Changes in the cell morphology were recorded by observing under phase-contrast inverted microscope (Olympus CK40-SLP) at 200X magnification. Trypan blue dye exclusion assay was used to confirm cell death. By this method, nonviable cells are stained whereas viable cells exclude the dye (33). Percent viability was determined as follows: [total number of viable cells per ml of aliquot/ total number of cells per ml of aliquot] × 100. The experiment was repeated three times and data were averaged. Standard error was calculated using the statistical software OriginPro^®^ version 9.9 freely available from https://www.originlab.com

#### Detection of Virulence Factor Encoding Genes by Polymerase Chain Reaction (PCR)

A total of 34 isolated strains were tested for the occurrence of four genetic determinants of virulence factors, *aer/haem* (haemolytic toxin), *aspA* (serine protease), *ascV* (type 3 secretion system), and *flaA* (polar flagella) by PCR using gene-specific primers (34). The primers have been listed in Supplementary Table 1. PCR amplification was performed with a 25 μl reaction volume containing 5 μl of 5X Flexi-Taq DNA polymerase buffer, 0.5 μl of 10 mM dNTP mix, 0.25 μl of the gene primers (100 mM-both forward and reverse), 2 μl of 25 mM MgCl_2_, 2 μl template DNA, and 0.25 μl of 5U Taq polymerase. PCRs were carried out in thermal cycler (Applied Biosystems GeneAmp PCR 2,400) with the following cycling conditions: initial denaturation at 95°C for 3 min, 30 cycles of denaturation at 95°C for 30 s, annealing at temperature of 50–55°C appropriate for each primer pair for 30 s, extension at 72°C for 30 s, and a final extension at 72°C for 5 min. The amplicons were separated electrophoretically in 2% agarose gel stained with ethidium bromide (0.5 μg/ml). Electrophoresis was performed in a tank containing 1X TAE buffer for 1 h at 55V and bands viewed under UV-transilluminator (Bio-Rad Laboratories). PCR products were extracted using Gel/PCR DNA Fragments Extraction Kit (BR Biochem Life Sciences Pvt. Ltd.) according to the supplier's protocol. The products were cloned using pGEM–T easy vector kit (PROMEGA Corporation, U.S.A.) following the manufacturer's instructions. The amplicons were sequenced at Eurofins sequencing services, Bangalore, India. The obtained sequences were subjected to the similarity search using the BLAST search program of the National Center for Biotechnology Information (NCBI) (35). The sequences obtained were annotated and thereafter deposited in the NCBI GenBank through the Bankit tool.

#### Conjugation and Plasmid Detection

The potential donor GP3 strain bearing the haemolytic gene and nalidixic acid-resistant *Escherichia coli-*DH5α recipient strain which tested negative in PCR with *aer/haem* primers was incubated overnight in LB broth at 37°C. Both were grown to equal optical densities of 0.5 as measured by spectrophotometer (10^7^ cells/ml). Broth conjugation was performed by mixing equal volumes of donor and recipient strains and incubating at 25°C for 24 h without shaking. A ten-fold serial dilution of each mating mixture was spread on LB agar plates supplemented with 5% sheep blood and 20 μg/ml nalidixic acid. Colonies growing on these double selective plates after 24–48 h of incubation at 37°C were treated as the putative transconjugants (32). The efficiency of conjugation was estimated as the number of transconjugants per initial number of recipients. Subsequently, boiling lysis of the selected transconjugants was carried out, and PCR was performed using *aer/heam* primers to detect the presence of the transferred gene. The donor and transconjugants as well as recipients prior to conjugation were also screened for the presence of plasmids as described by Birnboim and Doly (36).

## Results

### Occurrence and Presumptive Identification of *Aeromonas*

Out of the total 83 strains, 34 strains were morphologically and biochemically identified as *Aeromonas* sp. (Supplementary Table 2), and the identity was further confirmed by 16S rRNA gene sequencing. The genetic relationship among the sequences was established by a phylogenetic tree constructed by MEGA software (version 7) (Supplementary Figure 1). The neighbor-joining tree method showed that all the 34 isolates clustered with the reference strains of *Aeromonas* species: *A. veronii* (*n* = 19), *A. hydrophila* (*n* = 7), *A. cavie* (*n* = 3), and *Aeromonas jandei* (*n* = 5).

### Virulence Characteristics

The virulence characteristics of all isolates were studied, and the results are summarized in Supplementary Table 3. Haemolytic and proteolytic activity was a common trait exhibited by all the 34 (100%) isolates. On the other hand, 15 (44.1%), 13 (38.2%), and 24 (70.6%) isolated strains displayed DNase, siderophore, and amylase production, respectively. Only 2 (5.8%) isolates showed lipolytic activity.

### Pathogenicity in Fish

**Figure 1:**
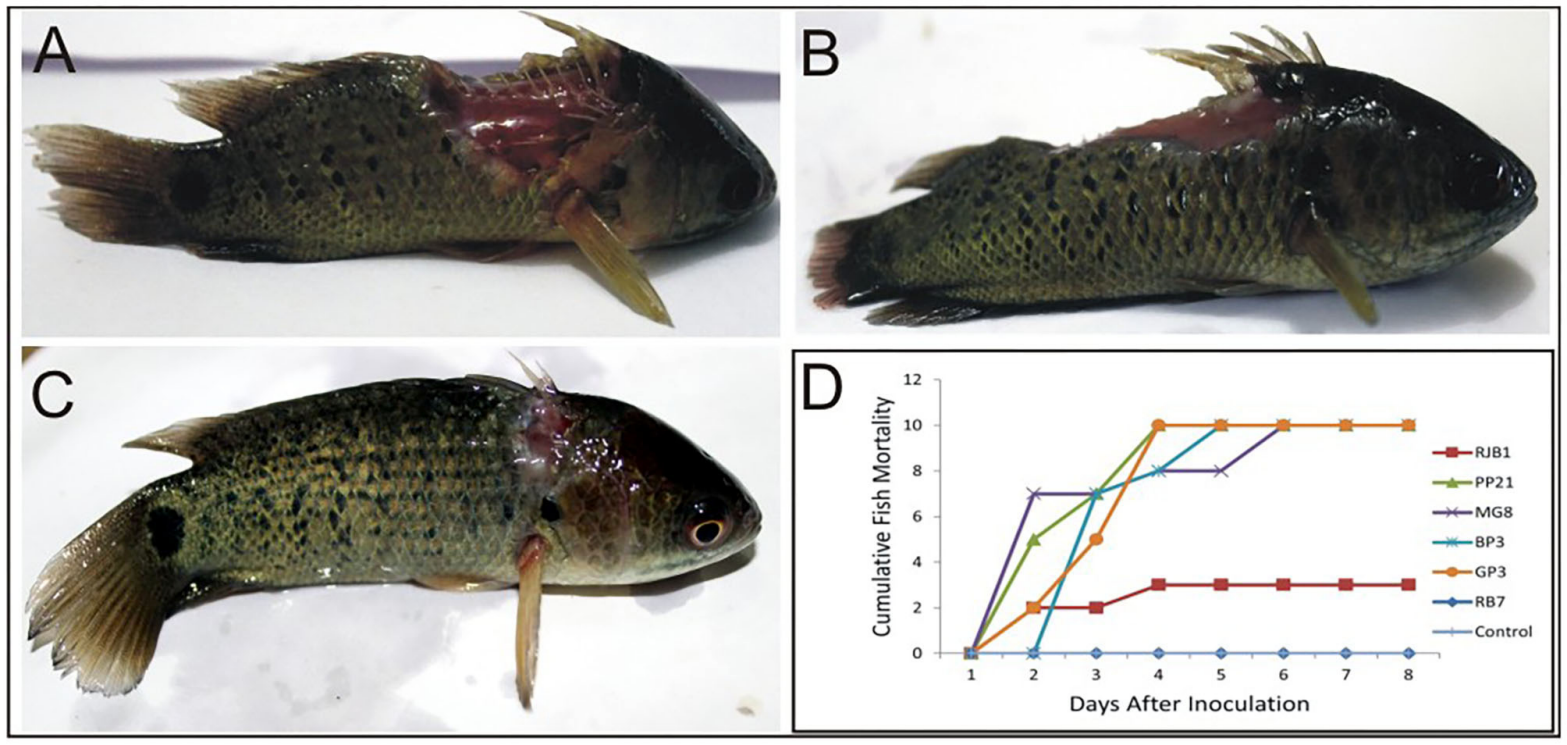
Pathogenicity testing of isolated *Aeromonas* strains in *A. testudineus*. **(A)** Extremely severe lesion in fish injected intramuscularly with cell suspension of GP3. **(B)** Fish injected with PP21 showing severe lesion. **(C)** Fish injected with RJB1showing superficial lesion. **(D)** Graph depicting cumulative mortality of fishes injected with bacterial cell suspensions within a 7-day observation period.

Virulence of six potential pathogenic *Aeromonas* strains isolated from different locations (GP3, RB7, BP3, RJB1, MG8, and PP21) was tested in *A. testidineus* using intramuscular injection, and the results are presented in Figure 1. Superficial lesions were observed initially at the site of injection in most fishes, which progressively became severe with time. Ulcers induced by GP3 were found to be most severe and all the fishes died by the 3rd day (Figure 1A). PP21-injected fishes also showed 100% mortality on the 3rd day with severe to moderate ulcers at the site of injection (Figure 1B). Fishes injected with BP3 and MG8 exhibited mild to moderate ulcers2 with 100% of mortality on 4th and 5th day, respectively (Figure 1D). RJB1-injected fishes produced superficial lesions in 30% of the fishes and recorded low mortality (Figure 1C). However, fishes injected with strain RB7 did not record any mortality. The fishes in the control group were healthy with no signs of ulcers or disease. The median survival time of fishes injected with PP21, MG8, BP3, and GP3 was found to be 1.5, 1, 2, and 2.5. For RJB1, RB7, and the control group, it was undefined because some fishes survived at the end of the observation period (Supplementary Figure 2). The results obtained were statistically significant with *p* < 0.0001 in the log-rank test.

### Cytotoxicity

**Figure 2:**
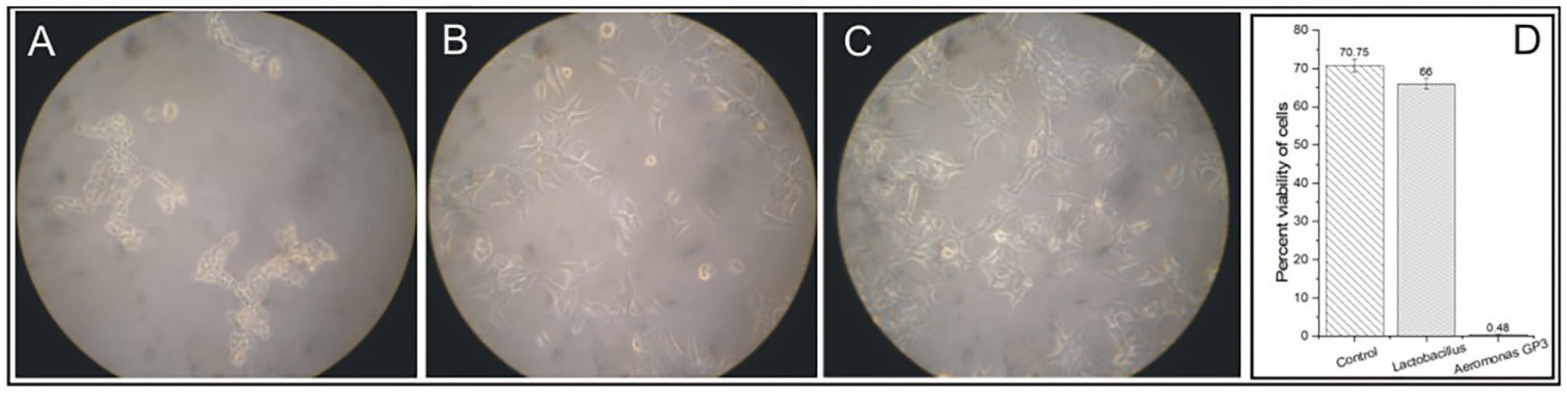
Cytotoxicity of isolated *Aeromonas* strain GP3 in WRL-68 cell line. **(A)** Treated with culture filtrate of GP3 showing visible rounding off and detachment of monolayers. **(B)** Treated with cell-free filtrate of *Lactobacillus* sp. showing minor morphological changes. **(C)** Control (treated with sterile LB medium) showing no change in cell morphology. **(D)** Graph representing the cell viability assay.

The cell-free culture filtrate of isolate GP3 induced cytotoxic activity on WRL-68 human liver cell lines. Degenerative changes such as visible rounding off and adherence loss from the plate surface in cell cultures were observed (Figure 2A). However, no visible changes in morphology were induced in cell lines exposed to *Lactobacillus* cell-free culture filtrates (Figure 2B) as well as in control (Figure 2C). In the cell viability assay, a significant reduction in percent viability was observed in the GP3-exposed cells when compared to the control. GP3-exposed cells recorded a percent viability of only 0.48% which was much lower than *Lactobacillus* treated cells (66%) (Figure 2D).

### Virulence Genes

The virulence genes were detected in 23 of the 34 isolates which were identified as *Aeromonas*. The *aerA/haem* and *flaA* gene were amplified in 44.11% of the isolates (Supplementary Figures 3A,B). The *ascV* and *aspA* gene were detected in 23.5 and 8.82% of the isolates, respectively (Supplementary Figures 3C,D). The isolates could be classified into eight groups depending upon the occurrence of virulence genes (Table 1). A total of eleven isolates did not show the presence of any of the virulence genes. The GenBank accession numbers of the virulence genes are given as *aer/haem* (MT704303-MT704309, MT707932-MT707935, MH607886, MT591426, and MTT813045) *aspA* (MT909568-MT909570), *ascV* (MW001219-MW001222, MH607887-MH607890), and *flaA* (MT942623-MT942626, MT977537- MT977539).

**Table 1:** *Aeromonas* isolates summarized into groups based on occurrence of virulence genes.

**Groups**	**Virulence related genes**				**Number of isolates (isolate names)**
	***aer/haem***	***aspA***	***ascV***	***flaA***	
A	+	–	+	+	5 (PP7, PP12, FP2, GP3, MG8)
B	+	+	–	+	1 (RJB1)
C	+	+	+	–	1 (RB7)
D	+	–	–	+	3 (PP21, PP22, FP8)
E	+	+	–	–	1 (BP6)
F	+	–	–	–	4 (HP1, HP6, BP2, GD3)
G	–	–	–	+	6 (SFM1, SFM2, PP19, PP23, GD1, RB5)
H	–	–	+	–	2 (BP3, GP1)
I	–	–	–	–	11
Total % of isolates	44.1%	8.82%	21.5%	44.1%	

*“+” indicates presence*.

*“–” indicates absence*.

### Conjugation and Plasmid Isolation

**Figure 3:**
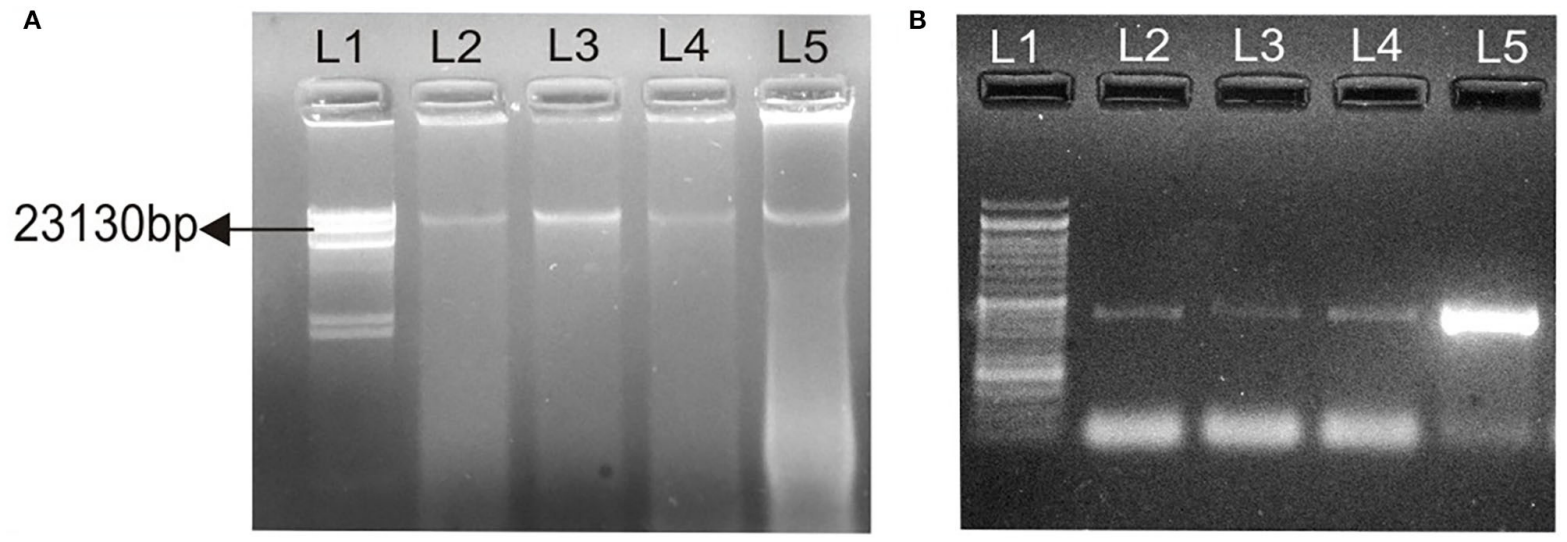
Transfer of virulence gene from isolated *Aeromonas* strain GP3 to *E. coli* DH5α. **(A)** lane 1, Lambda DNA/*Hind* III digest: molecular weight marker; lane 2, 23-kb plasmid (23,130 bp) of GP3 (donor); L3, L4, L5, 23 kb plasmids detected in transconjugants, **(B)** 431-bp PCR amplicons of the aerolysin/haemolysin (*aer/haem*) encoding gene from donor and transconjugants; lane 1, molecular weight marker as 50-bp DNA ladder; lanes 2-4, transconjugants and lane 5, donor GP3.

Conjugation experiments revealed that determinants of haemolytic property of the isolated *Aeromonas* strain GP3 was transferable to recipient *E. coli*. GP3 contained a 23-kb plasmid which was also detectable in the transconjugants (Figure 3A). PCR analysis revealed the presence of the 431bp *aer/haem* gene in both donor and transconjugants (Figure 3B). The gene was not detected in recipient strains prior to conjugation. The frequency of conjugal transfer was recorded as 0.394 × 10^−4^ transconjugants per recipient cell in a mating mixture.

## Discussion

In this study, several environmental strains of *Aeromonas* sp. harboring multiple virulence encoding genes were isolated from water samples of 10 fish-farming sites distributed across three districts of the Sub-Himalayan West Bengal. Furthermore, few isolates proved to be pathogenic in fish and cytotoxic to mammalian liver cells. Conjugational studies on the transfer of haemolysin-encoding gene from a virulent strain to *E. coli* Dh5α were also successfully demonstrated.

During the process of isolation, a lot of background microflora with similar cultural characteristics and morphology was obtained on *Aeromonas* isolation medium (37). Furthermore, we accurately identified the strains belonging to this genus using the aero key scheme proposed by Carnahan et al. (22) followed by 16S rRNA gene sequencing (38). Phylogenetic tree showed that all 34 isolates rearranged between four reference species with a highest number of species clustering to *A. veronii* followed by *A. hydrophila*. Few isolates formed distinct clades with *A. jandaei* and three isolates clustered with *A. caviae*. Such prevalence of variable species from the area of our study has not yet been reported elsewhere.

The foothill of Sub-Himalayan region is a geographically diverse region and serves as a habitat for a lot of endemic species of fish (39). A huge impact of bacterial diseases on fish productivity (40) and the role of *Aeromonas* in fish infections due to multiple virulence factors has been previously reported (41). In this study, all the strains isolated from water samples of small fish farms were found to be beta-haemolytic and proteolytic. This supports the theory of close association between the two factors since proteases have a prominent role in cleavage and activation of haemolysin (42). Additionally, 70.6% of the strains produced amylase whereas lesser isolates produced siderophore and DNase. Lower detection of siderophore-producing bacteria in this study may be due to alternate iron acquisition mechanisms found in *Aeromonas* for utilization in the process of host colonization (43). Likewise, DNase and amylase production are less as it depends on the nutritional requirement of the pathogen (42, 44).

In our study, four important virulence-related genes encoding haemolysin (*aerA/haem*), alkaline serine protease (*aspA)*, inner component of type III secretion system (*ascV)*, and polar flagella (*flaA)* had a heterogenous distribution among the isolates. Among these four genes, a prevalence of at least one virulence-related gene was observed in 67.7% of the total isolates. A higher percent (44.1%) of strains was found to harbor the *aer/haem* and *flaA* genes, which was similar to the findings by other authors (45, 46). However, lesser proportion of strains showed the presence of *ascV* (21.5%) and *aspA* (8.82%), indicating the possible involvement in proteases other than serine protease and secretion systems other than type III (47). *Aeromonas* isolates harboring at least one virulence gene were summarized into groups based on the presence of the type of gene. A total of eight different combinations were revealed, of which, the presence of single *flaA* gene, found in six isolates, was the most common. A total of five isolates showed the virulence gene pattern ae*r*/*haem*^+^/*ascV*^+^/*flaA*^+^. The *aerA* (encoding aerolysin) and *hlyA* (encoding haemolysin) gene in *Aeromonas hydrophila* has been previously categorized as strong virulence determinant for pathogenicity (48, 49). Li et al. (12) indicated a strong link between bacterial virulence and the presence of *aerA* and *ahp* (encoding serine protease) gene. Hence, in *Aeromonas*, the presence of multiple virulence factors encoding genes either acting singly or in a synergistic pattern leads to disease development in the host (46).

In the fish challenge studies, all the six selected isolates of *Aeromonas* were found to be pathogenic to *A. testudineus*. Mortality was reported in 72.2% of fishes along with lesion development at the site of administration of bacteria. An isolate GP3 exhibiting five virulence characteristics and harboring three of the four tested virulence-related genes showed a highest pathogenicity with severe lesions leading to 100% of mortality. PP21, with only two virulent phenotype (protease and haemolysin) and genes *aer/haem* and *flaA*, also showed 100% of mortality on the 3rd day following injection. On the other hand, RB7 which exhibited four virulent phenotypes (haemolysin, protease, DNase, and amylase) and three genes (*aer/haem*^+^*/ascV*^+^*/aspA*^+^) did not record any mortality. Further, BP3 which showed four virulence traits and harbored only one of the four tested genes (*ascV*) was found to be strongly pathogenic to fishes. Therefore, no distinct correlation among the exhibition of virulence phenotypes, harboring of specific genes, and the mortality in fishes was evident. Similar results were obtained by Oliviera et al. (50) as they found no statistically significant relationship between the presence of virulence factors and mortality in Nile tilapia infected by *A. hydrophila*. Such absence of correlation between virulence factors and pathogenicity has also been reported by many other researchers (51, 52). However, several authors observed that the presence of specific virulence factors in aeromonads is closely related to mortality in injected fishes (9, 53).

The virulence of GP3 was further evaluated by assessing its ability to induce cytotoxic changes in liver cell lines in comparison with a non-virulent strain *Lactobacillus*. In our study, clear rounding off and detachment of monolayers were visible in liver cells exposed to cell-free GP3 filtrate. The cell viability reduced to only 0.48%, indicating extreme cytotoxicity. In similar studies, previous authors have reported rounding off, shriveling, and detachment of cell lines treated with bacterial filtrates of *Aeromonas* (54). The cytotoxicity in WRL-68 human liver cell lines indicated the possibility of disease-causing ability of *Aeromonas* in human, and similar reports suggesting its role in gastroenteritis and soft tissue infections of humans have been reported earlier (2).

Another significant finding of our study is the successful transfer of a component of haemolytic gene *via* conjugation from a virulent isolate GP3 to an *E. coli* recipient strain. A 23-kb plasmid was isolated from donor and transconjugants. Previous studies demonstrating the transfer of virulence genes from other genera such as *Vibrio* and *S. aureus* have been reported (15, 16, 18), but such reports involving aeromonads are rare. Most of the reports in *Aeromonas* sp. have their prime focus on the distribution of genetic determinants of several virulence factors, but there is very less evidence to suggest the transfer ability of these genes to non-pathogenic strains of bacteria belonging to a same or different genus. Majumdar et al. (55) observed that curing of a particular 21-kb plasmid detected in virulent *Aeromonas* isolate from environmental sources led to the loss in virulence. In this study, the 23-kb plasmid may be carrying the haemolysin gene as it was isolated from donor and transconjugants but not from the recipient strain prior to conjugation. Such mobile genetic elements contribute to the gene pool in aquatic reservoirs (56) and could be easily transferred to other microorganisms helping them thrive in nutrient-limiting conditions and increase their survivability within hosts (57).

In conclusion, this study revealed the occurrence of virulence factors and some of their genetic determinants in *Aeromonas* strains of aquatic origin. Further extensive studies on a greater number of virulence genes shall serve to accurately discriminate the various pathotypes of *Aeromonas* spp. We further studied the probability of horizontal spread of haemolytic property from the isolated *Aeromonas* to *E. coli* recipient strain. To our knowledge, this is the first report of the transfer of *aer/haem* gene encoding a haemolysin from a strain of virulent *Aeromonas* to any recipient strain. As the aquatic bodies provide a perfect environment for the interaction of microbes leading to gene transfer among cohabitant microflora, the occurrence of transferable virulence traits in bacteria especially in fish farming habitats is alarming. Being opportunistic pathogens, these bacteria may also infect humans as indicated by cytotoxicity studies. Future studies on the involvement of mobile genetic elements in virulence of *Aeromonas* spp. from aquaculture sources will allow to accurately assess the risk of spread of virulence traits to other non-pathogenic bacteria and the threat this poses to public health. Therefore, periodic monitoring of *Aeromonas* is required as these serve as the indicators of poor fish culture conditions and would possibly lead to zoonotic outbreaks.

## Data Availability Statement

The datasets presented in this study can be found in online repositories. The names of the repository/repositories and accession number(s) can be found in the article/Supplementary Material.

## Ethics Statement

Ethical review and approval were not required as the study is in accordance with the institutional requirements and the institution has reviewed and approved the animal studies (Approval no. 161-(A)/R-2022). The fish used here is marketed in live condition commercially for purpose of human consumption. However, all fish handling and experimentation was done with maximum veterinary care.

## Author Contributions

DS designed the study, helped in interpreting the study results, and critically revised the manuscript. PM and PB carried out the methodology, collected, and analysed the data. PM and DS drafted the manuscript. AS has helped in fish studies. AK has contributed in cytotoxicity studies. All authors contributed to the article and approved the submitted version.

## Funding

This work was supported by funds from University Grants Commission (UGC), India (www.ugc.ac.in/) Grant No. F. No. 42-187/2013(SR) dated 02.07.2014. PM received fellowship from University Grants Commission (UGC), India (www.ugc.ac.in/) Grant No. F.25-1/2014-15(BSR)/7-132/2007(BSR) dated 07.10.2015. PB received fellowship from Department of Biotechnology, Govt. of India. The funders had no role in study design, data collection and analysis, decision to publish, or provide any funds for publishing.

## Conflict of Interest

The authors declare that the research was conducted in the absence of any commercial or financial relationships that could be construed as a potential conflict of interest.

## Publisher's Note

All claims expressed in this article are solely those of the authors and do not necessarily represent those of their affiliated organizations, or those of the publisher, the editors and the reviewers. Any product that may be evaluated in this article, or claim that may be made by its manufacturer, is not guaranteed or endorsed by the publisher.
