# Supplementary material for: Detection of Virulence-Associated Genes and in vitro Gene Transfer From Aeromonas sp. Isolated From Aquatic Environments of Sub-himalayan West Bengal
Source: Front Vet Sci. 2022 Jun 10;9:887174. doi: 10.3389/fvets.2022.887174 (PMC9230572; doi:10.3389/fvets.2022.887174)
Supplement: Supplementary file 1 [file Table_1.DOCX]

Supplementary Material

**Supplementary Table 1: List of virulence related gene primers used in study**

| **Gene** | **Protein product** | **Sequence (5’-3’)** | **Annealing temperature(°C)** | **Reference** |
| --- | --- | --- | --- | --- |
| *aerA/haem* | Haemolysin | F:CCTATGGCCTGAGCGAGAAG | 55.3°C | Roman *et al*.,2014 |
|  |  | R:CCAGTTCCAGTCCCACCACT- |  |  |
| *ascV* | Inner component of typeIII secretion system | F:ATGGACGGCGCCATGAAGTT | 55.5°C |  |
|  |  | R:TATTCGCCTTCACCCATCCC |  |  |
| *aspA* | Alkaline serine protease | F: CACCGAAGTATTGGGTCAGG | 53.3°C |  |
|  |  | R: GGCTCATGCGTAACTCTGGT |  |  |
| *flaA* | Flagellin | F: TCCAACCGTYTGACCTC | 50.2 |  |
|  |  | R: GMYTGGTTGCGRATGGT |  |  |

**Supplementary Table 2. *Aeromonas* strains isolated from water samples of three districts of West Bengal**

| **Place of sampling** | **District/state** | **Latitude/longitude** | **Codes assigned** | **Total no.of isolates** | ***Aeromonas* isolates** |
| --- | --- | --- | --- | --- | --- |
| Shivmandir | Darjeeling/ West Bengal | 26°42'35.04"N/88°21'27.40"E | SM | 6 | SM1, SM2, SM5 |
| Paharpura | Jalpaiguri/ West Bengal | 26°34'23.17"N/88°43'31.27"E | PP | 25 | PP7, PP12, PP13, PP19, PP21, PP22, PP23 |
| Heliopakhri | Jalpaiguri/ West Bengal | 26°25'37.80"N/88°51'39.20"E | HP | 7 | HP1, HP6 |
| Baruapara | Jalpaiguri/ West Bengal | 26°32'45.97"N/88°39'2.10"E | BP | 9 | BP2, BP3, BP5, BP6 |
| Gazaldoba | Jalpaiguri/ West Bengal | 26°45'57.12"N/88°36'24.52"E | GD | 4 | GD1GD3 |
| **Place of sampling** | **District/state** | **Latitude/longitude** | **Codes assigned** | **Total no.of isolates** | ***Aeromonas* isolates** |
| Fatapukur | Jalpaiguri/ West Bengal | 26°34'11.64"N/88°32'24.91"E | FP | 8 | FP2, FP5, FP8 |
| RautBagan | Jalpaiguri/ West Bengal | 26°29'5.56"N/88°41'52.00"E | RB | 7 | RB2, RB4, RB5, RB7 |
| Gopalpurhat | Jalpaiguri/ West Bengal | 24° 5'26.09"N/88°41'8.78"E | GP | 4 | GP1, GP3 |
| Mekhliganj | Cooch Behar/ West Bengal | 26°20'50.60"N/88°54'36.90"E | MG | 8 | MG3, MG6, MG8, MG9 |
| Rajbari | Cooch Behar/ West Bengal | 26°19'38.29"N/89°26'17.67"E | RJB | 5 | RJB1, RJB3, RJB5 |
|  |  | Total no. of isolates |  | 83 | 34 |

**Supplementary Table 3: Screening of the *Aeromonas* isolates for the presence of extracellular virulence factors**

| **Strains** | **Virulence factors** | | | | | |
| --- | --- | --- | --- | --- | --- | --- |
|  | **Haemolysin** | **Protease** | **Lipase** | **DNase** | **Siderophore** | **Amylase** |
| SM1 | + | + | - | - | - | - |
| SM2 | + | + | - | - | - | - |
| SM5 | + | + | - | - | - | - |
| PP7 | + | + | - | + | + | + |
| PP12 | + | + | - | + | - | + |
| PP13 | + | + | - | - | - | - |
| PP19 | + | + | - | + | + | + |
| **Strains** | **Virulence factors** | | | | | |
|  | **Haemolysin** | **Protease** | **Lipase** | **DNase** | **Siderophore** | **Amylase** |
| PP21 | + | + | - | - | - | - |
| PP22 | + | + | - | + | + | + |
| PP23 | + | + | - | - | - | - |
| HP1 | + | + | - | - | + | + |
| HP6 | + | + | - | - | + | + |
| BP2 | + | + | - | + | + | + |
| BP3 | + | + | - | + | - | + |
| BP5 | + | + | - | + | + | + |
| BP6 | + | + | - | - | - | + |
| GD1 | + | + | - | - | - | + |
| GD3 | + | + | - | - | - | - |
| FP2 | + | + | - | + | + | + |
| FP5 | + | + | + | - | - | - |
| FP8 | + | + | - | + | + | + |
| RB2 | + | + | - | - | - | - |
| RB4 | + | + | - | + | + | + |
| RB5 | + | + | - | - | - | - |
| RB7 | + | + | - | + | - | + |
|  | **Virulence factors** | | | | | |
|  | **Haemolysin** | **Protease** | **Lipase** | **DNase** | **Siderophore** | **Amylase** |
| GP1 | + | + | - | + | - | + |
| GP3 | + | + | + | - | + | + |
| MG3 | + | + | - | + | - | + |
| MG6 | + | + | - | + | - | + |
| MG8 | + | + | - | + | - | + |
| MG9 | + | + | - | - | - | + |
| RJB1 | + | + | - | - | + | + |
| RJB3 | + | + | - | - | - | + |
| RJB5 | + | + | - | - | + | + |

**‘+’ indicates positive**

**‘-’ indicates negative**

**Supplementary figures**

**
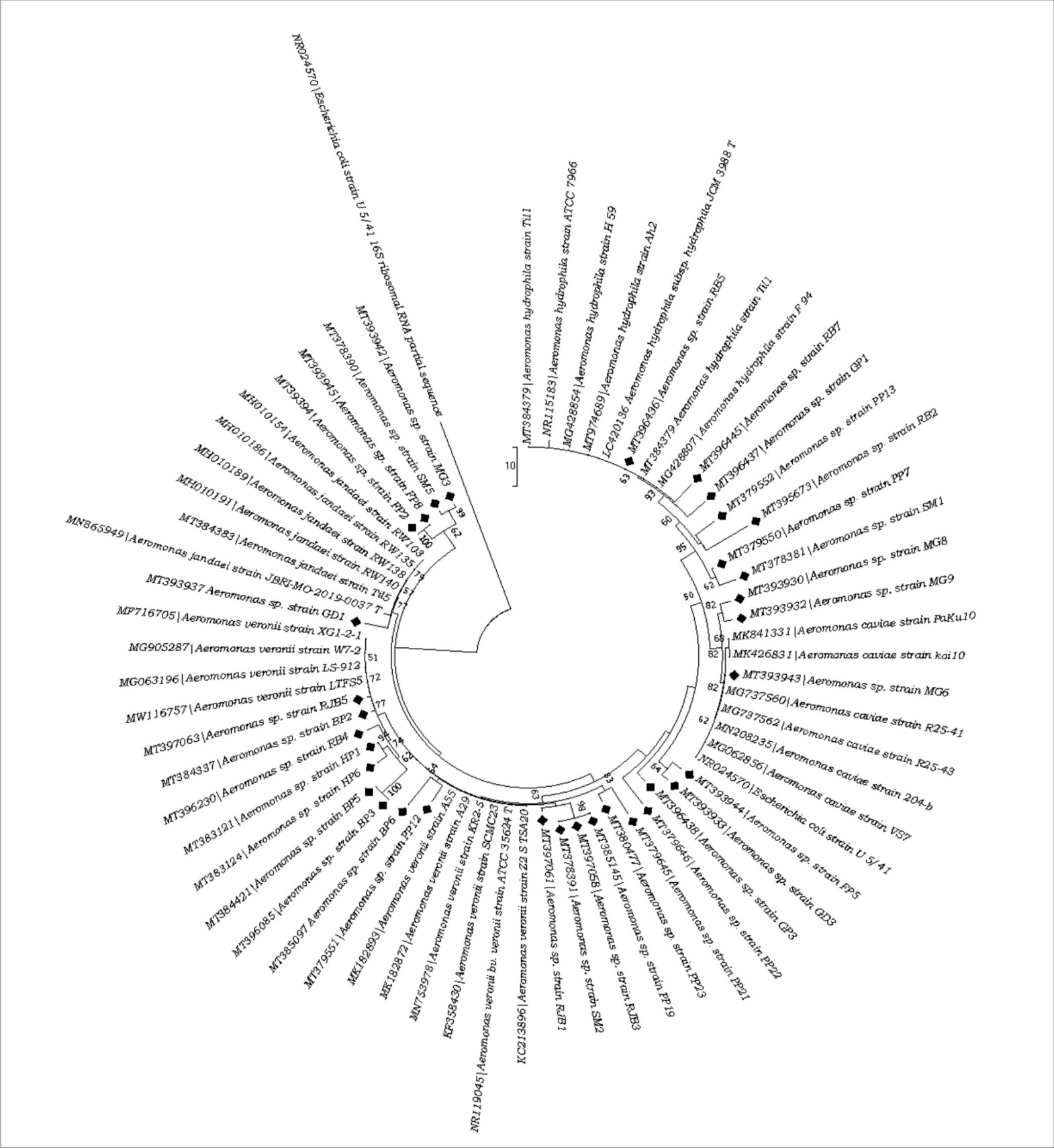
**

**Supplementary Figure 1.** Phylogenetic tree generated by neighbour-joining method on the basis of partial 16S rRNA gene sequences of the 34 isolates of *Aeromonas* used in this study. Bootstrap value expressed as percentages of 1000 replications as shown at nodes. The marked strains are the isolates of the present study.


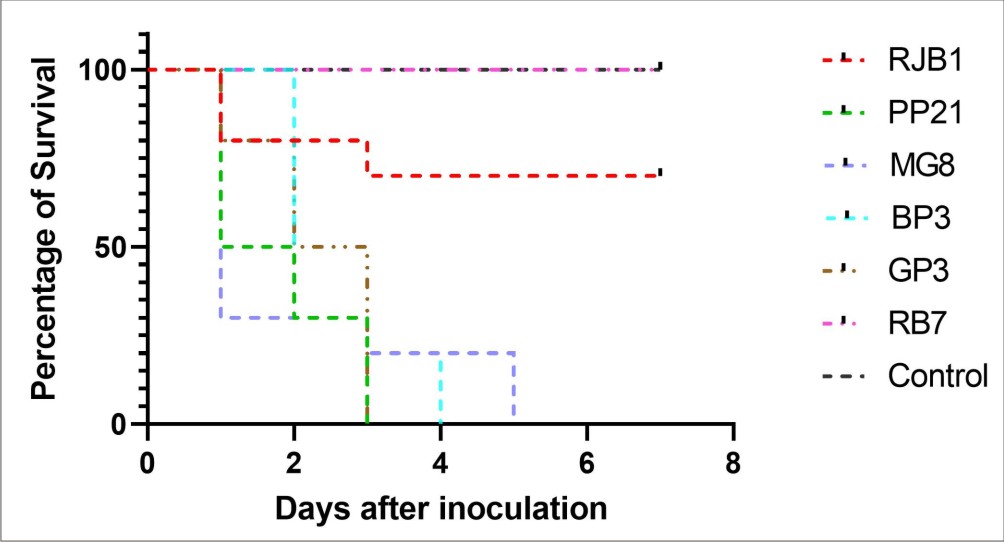


**Supplementary Figure 2.** Kaplan- Meier survival curve for pathogenicity testing of the six bacterial isolates in fishes.


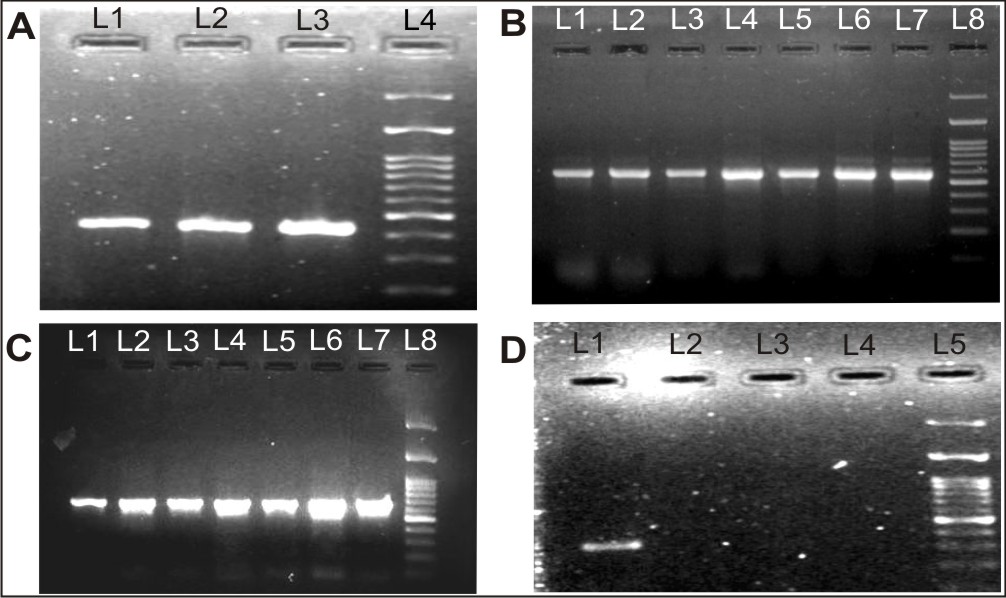


**Supplementary Figure 3.** Amplification of virulence associated genes from *Aeromonas* isolates **(A)** Lanes1-3: Agarose gel showing 431 bp PCR amplicons of the aerolysin/haemolysin (*aer/haem*) encoding gene from various isolates, Lane 4: DNA molecular weight marker (100 bp step up ladder) **(B)** Lanes1-7: 608 bp PCR amplicons of the polar flagella (*flaA)* encoding gene; Lane 8: 100 bp step up ladder; **(C)** Lanes1-7: 710 bp PCR amplicons of *ascV* gene encoding inner component of the type III secretion system, Lane 8: 100 bp stepup ladder (**D)** Lane 1: 350 bp PCR amplicon of the *aspA* gene encoding alkaline serine protease , Lane 5: 100 bp step up ladder.

**Genomic DNA Sequences**

**MT378381**

TGCAATTGAGATTTGATCATGGCTCAGATTGAACGCTGGCGGCAGGCCTAACACATGCAAGTCGAGCGGC

AGCGGGAAAGTAGCTTGCTACTTTTGCCGGCGAGCGGCGGACGGGTGAGTAATGCCTGGGAAATTGCCCA

GTCGAGGGGGATAACAGTTGGAAACGACTGCTAATACCGCATACGCCCTACGGGGGAAAGCAGGGGACCT

TCGGGCCTTGCGCGATTGGATATGCCCAGGTGGGATTAGCCAGTTGGTGAGGTAATGGCTCGCCAAGGCG

ACGATCCCTAGCTGGTCTGAGAGGATGATCAACCACACTGGAACTGAGACACGGTCCAGACTCCTGCGGG

AGGCAGCAGTGGGGAATATTGCACAATGGGGGAAACCCTGAAGCAGCCATGCCGCGTGTGTGAAGAAGGC

CTTCGGATTGTAAAGCACTTTCAGCGAGGAGGAAAGGTTGATGCCCAATACGTATCAACTGTGACGTTAC

TCGCAGAAGAAGCACCGGCTAACTCCGTGCCAGCAGCCGCGGTAATACGAGGGTGCAAGCGTTAATCGGA

ATTACTGGGCGTAAAGCGCACGCAGGCGGTTGGATAAGTTAGATGTGAAAGCCCCGGGCTCAACCTGGGA

ATTGCATTTAAAACTGTCCAGCTAGAGTCTTGTAGAGGGGGGTAGAATTCCAGGTGTAGCGGTGAAATGC

GTAGAGATCTGGAGGAATACCGGTGGCGAAGGCGGCCCCCTGGACAAAGACTGACGCTCAGGTGCGAAAG

CGTGGGGAGCAAACAGGATTAGATACCCTGGTAGTCCACGCCGTAAACGATGTCGATTTGGAGGCTGTGT

CCTTGAGACGTGGCTTCCGGAGCTAACGCGTTAAATCGACCGCCTGGGGAGTACGGCCGCAAGGTTAAAA

CTCAAATGAATTGACGGGGGCCCGCACAAGCGGTGGAGCATGTGGTTTAATTCGATGCAACGCGAAGAAC

CTTACCTGGCCTTG

**MT378391**

TATGCGTTGCGCAGCCTTCCCATGCAAGTCCGAGCGGCAGCGGGAAAGTTAGCTTTGCTACTTTTTGCCG

GCGAGCGGCGGACGGGTGAGTAATGCCTGGGGATCTGCCCAGTCGAGGGGGATAACTACTGGAAACGGTA

GCTAATACCGCATACGCCCTACGGGGGAAAGCAGGGGACCTTCGGGCCTTGCGCGATTGGATGAACCCAG

GTGGGATTAGCTAGTTGGTGAGGTAATGGCTCACCAAGGCGACGATCCCTAGCTGGTCTGAGAGGATGAT

CAGCCACACTGGAACTGAGACACGGTCCAGACTCCTACGGGAGGCAGCAGTGGGGAATATTGCACAATGG

GGGAAACCCTGATGCAGCCATGCCGCGTGTGTGAAGAAGGCCTTCGGGTTGTAAAGCACTTTCAGCGAGG

AGGAAAGGTTGGTAGCTAATAACTGCCAGCTGTGACGTTACTCGCAGAAGAAGCACCGGCTAACTCCGTG

CCAGCAGCCGCGGTAATACGGAGGGTGCAAGCGTTAATCGGAATTACTGGGCGTAAAGCGCACGCAGGCG

GTTGGATAAGTTAGATGTGAAAGCCCCGGGCTCAACCTGGGAATTGCATTTAAAACTGTCCAGCTAGAGT

CTTGTAGAGGGGGGTAGAATTCCAGGTGTAGCGGTGAAATGCGTAGAGATCTGGAGGAATACCGGTGGCG

AAGGCGGCCCCCTGGACAAAGACTGACGCTCAGGTGCGAAAGCGTGGGGAGCAAACAGGATTAGATACCC

TGGTAGTCCACGCCGTAAACGATGTCGATTTGGAGGCTGTGTCCTTGAGACGTGGCTTCCGGAGCTAACG

CGTTAAATCGACCGCCTGGGGAGTACGGCCGCAAGGTTAAGACTCAAATGAATTGACGGGGGCCCGCACA

AGCGGTGGAGCATGTGGTTTAATTCAATGCAACGCGAAGAACCTTACCTGGCCTTGACATGTCTGGAATC

CTGTAAAGATACGGGAGTGCCTTCGGGAATCAGAACACAGGTGCTGCATGGCTGTCGTCAGCTCGTGTCG

TGAGATGTTGGGTTAAGTCCCGCAACGAGCGCAACCCCTGTCCTTTGTTGCCAGCACGTAATGGTGGGAA

CTCAAGGGAGACTGCCGGTGATAAACCGGAGGAAGGTGGGGATGACGTCAAGTCATCATGGCCCTTACGG

CCAGGGCTACACACGTGCTACAATGGCGCGTACAGAGGGCTGCAAGCTAGCGATAGTGAGCGAATCCCAA

AAAGCGCGTCGTAGT

**MT378390**

GGCTGGCTGGGCTACACATGCAGTCGAGCGGCAGCGGGAAAGTAGCTTGCTACTTTTGCCGGCGAGCGGC

GGACGGGTGAGTAATGCCTGGGAAATTGCCCAGTCGAGGGGGATAACAGTTGGAAACGACTGCTAATACC

GCATACGCCCTACGGGGGAAAGCAGGGGACCTTCGGGCCTTGCGCGATTGGATATGCCCAGGTGGGATTA

GCTAGTTGGTGAGGTAATGGCTCACCAAGGCGGACGATCCCTAGCTGGTCTGAGAGGATGATCAGCCACA

CTGGAACTGAGACACGGTCCAGACTCCTACGGGAGGCAGCAGTGGGGAATATTGCACAATGGGGGAAACC

CTGATGCAGCCATGCCGCGTGTGTGAAGAAGGCCTTCGGGTTGTAAAGCACTTTCAGCGAGGAGGAAAGG

TTAGTACCTAATATCTGCTGGCTGTGACGTTACTCGCAGAAGAAGCACCGGCTAACTCCGTGCCAGCAGC

CGCGGTAATACGGAGGGTGCAAGCGTTAATCGGAATTACTGGGCGTAAAGCGCACGCAGGCGGTTGGATA

AGTTAGATGTGAAAGCCCCGGGCTCAACCTGGGAATTGCATTTAAAACTGTCCAGCTAGAGTCTTGTAGA

GGGGGGGCAGAATTTCCAGGTGTAGCGGTGAAATGCGTAGAGATCTGGAGGAATACCGGTGGCGAAGGCG

GCCCCCTGGACAAAGACTGACGCTCAGGTGCGAAAGCGTGGGGAGCAAACAGGATTAGATACCCTGGTAG

TCCACGCCGTAAACGATGTCGATTTGGAGGCTGTGTCCTTGAGACGTGGCT

**MT379550**

TGCAAGTCGAGCGGCAGCGGGAAAGTAGCTTGCTACTTTTGCCGGCGAGCGGCGGACGGGTGAGTAATGC

CTGGGAAATTGCCCAGTCGAGGGGGATAACAGTTGGAAACGACTGCTAATACCGCATACGCCCTACGGGG

GAAAGCAGGGGACCTTCGGGCCTTGCGCGATTGGATATGCCCAGGTGGGATTAGCTAGTTGGTGAGGTAA

TGGCTCACCAAGGCGACGATCCCTAGCTGGTCTGAGAGGATGATCAGCCACACTGGAACTGAGACACGGT

CCAGACTCCTACGGGAGGCAGCAGTGGGGAATATTGCACAATGGGGGAAACCCTGATGCAGCCATGCCGC

GTGTGTGAAGAAGGCCTTCGGGTTGTAAAGCACTTTCAGCGAGGAGGAAAGGTTGATGCCTAATACGTAT

CAACTGTGACGTTACTCGCAGAAGAAGCACCGGCTAACTCCGTGCCAGCAGCCGCGGTAATACGGAGGGT

GCAAGCGTTAATCGGAATTACTGGGCGTAAAGCGCACGCAGGCGGTTGGATAAGTTAGATGTGAAAGCCC

CGGGCTCAACCTGGGAATTGCATTTAAAACTGTCCAGCTAGAGTCTTGTAGAGGGGGGTAGAATTCCAGG

TGTAGCGGTGAAATGCGTAGAGATCTGGAGGAATACCGGTGGCGAAGGCGGCCCCCTGGACAAAGACTGA

CGCTCAGGTGCGAAAGCGTGGGGAGCAAACAGGATTAGATACCCTGGTAGTCCACGCCGTAAACGATGTC

GATTTGGAGGCTGTGTCCTTGAGACGTGGCTTCCGGAGCTAACGCGTTAAATCGACCGCCTGGGGAGTAC

GGCCGCAAGGTTAAACTCAAATGAATTGACGGGGGCCCGCACAAGCGGTGGAGCATGTGGTTTATTCGAT

GCACGCGAGAACCTTACTGGCCTGACTGGCTGGAATCTGC

**MT379551**

TTAGAGTTTGAATCCTGGCTCAGATTGAACGCTGGCGGGAGGCCTAACACATGCAAGTCGAGCGGCAGCG

GGAAAGTAGCTTGCTACTTTTGCCGGCGAGCGGCGGACGGGTGAGTAATGCCTGGGGATCTGCCCAGTCG

AGGGGGATAACTACTGGAAACGGTAGCTAATACCGCATACGCCCTACGGGGGAAAGCAGGGGACCTTCGG

GCCTTGCGCGATTGGATGAACCCAGGTGGGATTAGCTAGTTGGTGAGGTAATGGCTCACCAAGGCGACGA

TCCCTAGCTGGTCTGAGAGGATGATCAGCCACACTGGAACTGAGACACGGTCCAGACTCCTACGGGAGGC

AGCAGTGGGGAATATTGCACAATGGGGGAAACCCTGATGCAGCCATGCCGCGTGTGTGAAGAAGGCCTTC

GGGTTGTAAAGCACTTTCAGCGAGGAGGAAAGGTTGGTAGCTAATAACTGCCAGCTGTGACGTTACTCGC

AGAAGAAGCACCGGTTAACTCCGTGCCAGCAGCCGCGGTAATACGGAGGGTGCAAGCGTTAATCGGAATT

ACTGGGCGTAAAGCGCACTCAGGCGGTTGGATAAGTTAGATGTGAAAGCCCCGGGCTCAACCTGGGAATT

GCATTTAAAACTGTCCAGCTAGAGTCTTGTAGAGGGGGGTAGAATTCCAGGTGTAGCGGTGAAATGCGTA

GAGATCTGGAGGAATACCGGTGGCAAAGGCGGCCCCCTGGACCAAAGACTGACGCTCAGGGGCGAAAAGC

GTGGGAGCAACCAGATAA

**MT379552**

GGCGGCTACACATGCAAGTCGAGCGGCAGCGGGAAAGTAGCTTGCTACTTTTGCCGGCGAGCGGCGGACG

GGTGAGTAATGCCTGGGAAATTGCCCAGTCGAGGGGGATAACAGTTGGAAACGACTGCTAATACCGCATA

CGCCCTACGGGGGAAAGCAGGGGACCTTCGGGCCTTGCGCGATTGGATATGCCCAGGTGGGATTAGCTAG

TTGGTGAGGTAATGGCTCACCAAGGCGACGATCCCTAGCTGGTCTGAGAGGATGATCAGCCACACTGGAA

CTGAGACACGGTCCAGACTCCTACGGGAGGCAGCAGTGGGGAATATTGCACAATGGGGGAAACCCTGATG

CAGCCATGCCGCGTGTGTGAAGAAGGCCTTCGGGTTGTAAAGCACTTTCAGCGAGGAGGAAAGGTTGATG

CCTAATACGTATCAACTGTGACGTTACTCGCAGAAGAAGCACCGGCTAACTCCGTGCCAGCAGCCGCGGT

AATACGGAGGGTGCAAGCGTTAATCGGAATTACTGGGCGTAAAGCGCACGCAGGCGGTTGGATAAGTTAG

ATGTGAAAGCCCCGGGCTCAACCTGGGAATTGCATTTAAAACTGTCCAGCTAGAGTCTTGTAGAGGGGGG

TAGAATTCCAGGTGTAGCGGTGAAATGCGTAGAGATCTGGAGGAATACCGGTGGCGAAGGCGGCCCCCTG

GACAAAGACTGACGCTCAGGTGCGAAAGCTTGGGGAACAAACAGGATTAGATACCCCGGTATTCCACGCC

GTAAACGATGTCTATTTGGAGGCTGGGTCCTTGAGACGTGGGTTCCGGAGCTAACGCGTTAAATCGACCG

CCTGGGGAGTACGGCCGCAAGGTTAAAACTCAAATGAATTGACGGGGGCCCGCACAAGCGGTGGAGCATG

TGGTTTAATTCGATGCAACGCGAAGAACCTTACCTGGCCTTGACATGTCTGGAATCCTGCAGAGATGCGG

GAGTGCCTTCGGGAATCAGAACACAGGTGCTGCATGGCTGTCGTCAGCTCGTGTCGTGAGATGTTGGGTT

AAGTCCCGCAACGAGCGCAACCCCTGTCCTTTGTTGCCAGCACGTAATGGTGGGAACTCAAGGGAGACTG

CCGGTGATAAACCGGAGGAAGGTGGGGATGACGTCAAGTCATCATGGCCCTTACGGCCAGGGCTACACAC

GTGCTACAATGGCGCGTACAGAGGGCTGCAAGCTAGCGATAGTGAGCGAATCCCAAAAAGCGCGTCGTAG

TCCGGATCGGAGTCTGCAACTCGACTCCGTGAAGTCGGAATCGCTAGTAATCGCAAATCAGAATGTTGCG

GTGAATACGTTCCCGGGCCTTGTACACACCGCCCGTCACACCATGGGAGTGGGTTGCACCAGAAGTAGAT

AGCTTAACCTTCGGGAGGGCGTTACCAGCGGTGATTC

**MT385145**

TATGCGTTGCGCAGCCTTCCCATGCAAGTCCGAGCGGCAGCGGGAAAGTTAGCTTTGCTACTTTTTGCCG

GCGAGCGGCGGACGGGTGAGTAATGCCTGGGGATCTGCCCAGTCGAGGGGGATAACTACTGGAAACGGTA

GCTAATACCGCATACGCCCTACGGGGGAAAGCAGGGGACCTTCGGGCCTTGCGCGATTGGATGAACCCAG

GTGGGATTAGCTAGTTGGTGAGGTAATGGCTCACCAAGGCGACGATCCCTAGCTGGTCTGAGAGGATGAT

CAGCCACACTGGAACTGAGACACGGTCCAGACTCCTACGGGAGGCAGCAGTGGGGAATATTGCACAATGG

GGGAAACCCTGATGCAGCCATGCCGCGTGTGTGAAGAAGGCCTTCGGGTTGTAAAGCACTTTCAGCGAGG

AGGAAAGGTTGGTAGCTAATAACTGCCAGCTGTGACGTTACTCGCAGAAGAAGCACCGGCTAACTCCGTG

CCAGCAGCCGCGGTAATACGGAGGGTGCAAGCGTTAATCGGAATTACTGGGCGTAAAGCGCACGCAGGCG

GTTGGATAAGTTAGATGTGAAAGCCCCGGGCTCAACCTGGGAATTGCATTTAAAACTGTCCAGCTAGAGT

CTTGTAGAGGGGGGTAGAATTCCAGGTGTAGCGGTGAAATGCGTAGAGATCTGGAGGAATACCGGTGGCG

AAGGCGGCCCCCTGGACAAAGACTGACGCTCAGGTGCGAAAGCGTGGGGAGCAAACAGGATTAGATACCC

TGGTAGTCCACGCCGTAAACGATGTCGATTTGGAGGCTGTGTCCTTGAGACGTGGCTTCCGGAGCTAACG

CGTTAAATCGACCGCCTGGGGAGTACGGCCGCAAGGTTAAGACTCAAATGAATTGACGGGGGCCCGCACA

AGCGGTGGAGCATGTGGTTTAATTCAATGCAACGCGAAGAACCTTACCTGGCCTTGACATGTCTGGAATC

CTGTAAAGATACGGGAGTGCCTTCGGGAATCAGAACACAGGTGCTGCATGGCTGTCGTCAGCTCGTGTCG

TGAGATGTTGGGTTAAGTCCCGCAACGAGCGCAACCCCTGTCCTTTGTTGCCAGCACGTAATGGTGGGAA

CTCAAGGGAGACTGCCGGTGATAAACCGGAGGAAGGTGGGGATGACGTCAAGTCATCATGGCCCTTACGG

CCAGGGCTACACACGTGCTACAATGGCGCGTACAGAGGGCTGCAAGCTAGCGATAGTGAGCGAATCCCAA

AAAGCGCGTCGTAGT

**MT379645**

TAGTGGCAGGCGCAGCTACCATGCAGTCGAGCGGCAGCGGGAAAGTAGCTTGCTACTTTTGCCGGCGAGC

GGCGGACGGGTGAGTAATGCCTGGGGATCTGCCCAGTCGAGGGGGATAACTACTGGAAACGGTAGCTAAT

ACCGCATACGCCCTACGGGGGAAAGCAGGGGACCTTCGGGCCTTGCGCGATTGGATGAACCCAGGTGGGA

TTAGCTAGTTGGTGAGGTAATGGCTCTGAAGGCGACGATCCCTAGCTGGTCTGAGAGGATGATCAGCCAC

ACTGGAACTGAGACACGGTCCAGACTCCTACGGGAGGCAGCAGTGGGGAATATTGCACAATGGGGGAAAC

CCTGATGCAGCCATGCCGCGTGTGTGAAGAAGGCCTTCGGGTTGTAAAGCACTTTCAGCGAGGAGGAAAG

GTTGGTAGCTAATAACTGCCAGCTGTGACGTTACTCGCACAAGAAGCACCGGCTAACTCCGTGCCAGCAG

CCGCGGTAATACGGAGGGTGCAAGCGTTAATCGGAATTACTGGGCGTAAAGCGCACGCAGGCGGTTGGAT

AAGTTAGATGTGAAAGCCCCGGGCTCAACCTGGGAATTGCATTTAAAACTGTCCAGCTAGAGTCTTGTAG

AGGGGAGTAGAATTCCAGGTGTAGCGGTGAAATGCGTAGAGATCTGGAGGAATACCGGTGGCGAAGGCGG

CCCCCTGGACAAAGACTGACGCTCAGGTGCGAAAGCGTGGGGAGCAAACATGATTATATACCCTGGTAGT

CCACGCCGTAAACGATGTCGATTTGGAGGCTGTGTCCTTGAGACGTGGCTTCCGGAGTCTAACGCGTTAA

ATCGACCGCCTGGGGAGTACGGCCGCAAAGTTAAAACTCAAATGATTGTACGGGGCCCCGCACAAGCGGT

GGAGCATGTGGTTTAATTCGATGCAACGCGAAGAGCCTTACCTGGCCTTGACATGTCTGGAATCCTGCAG

AGATGCGGGAGTGCCTTCGGGAATCAGAACACAGGTGCTGCATGGCTGTCGTCAGCTCGTGTCGTGAGAT

GTTGGGTTAAGTCCCGCAACGAGCGCAACCCCTGTCCTTTGTTGCCAGCACGTAATGGTGGGAACTCAAG

GGAGACTGCCGGTGATAAACCGGAGGAAGGTGGGGATGACGTCAAGTCATCATGGCCCTTACGGCCAGGG

CTACACACGTGCTACAATGGCGCGTACAGAGGGCTGCAAGCTAGCGATAGTGAGCGAATCCCAAAAAGCG

CGTCGTAGTCCGGATCGGAGTCTGCAACTCGACTCCGTGAAGTCGGAATCGCTAGTAATCGCAAATCAGA

ATGTTGCGGTGAATACGTTCCCGGGCCTTGTACACACCGCCCGTCACACCATGGGAGTGGGTTGCACCAG

AAGTAGATAGCTTAACCTTCGGGAGGGCGTACCAGCGGTATATGTCC

**MT379646**

CAACAGTAGTCAAGTCGAGCGGCAGCGGGAAAGTAGCTTGCTACTTTTGCCGGCGAGCGGCGTAAGGGTG

AGTAGTGCCTGGGGATCTGCCAAGTCGAGGGGGATAACTATTGGAAACGACTGCTAATACCGCATACGCC

CTACGGGGGAAAGCAGGGGACCTTCGGGCCTTGCGCGATTGGATGAACCCAGGTGGGATTAGCTAGTTGG

TGAGGTAATGGCTCACCAAGGCGACGATCCCTAGCTGGTCTGAGAGGATGATCAGCCACACTGGAACTGA

GACACGGTCCAGACTCCTACGGGAGGCAGCAGTGGGGAATATTGCACAATGGGGGAAACCCTGATGCAGC

CATGCCGCGTGTGTGAAGAAGGCCTTCGGGTTGTAAAGCACTTTCAGCGAGGAGGAAAGGTTGTTGGCTA

ATATCTGCCAGCTGTGACGTTACTCGCAGAAGAAGCACCGGCTAACTCCGTGCCAGCAGCCGCGGTAATA

CGGAGGGTGCAAGCGTTAATCGGAATTACTGGGCGTAAAGCGCACGCAGGCGGTTGGATAAGTTAGATGT

GAAAGCCCCGGGCTCAACCTGGGAATTGCATTTAAAACTGTCCAGCTAGAGTCTTGTAGAGGGGGGTAGA

ATTCCAGGTGTAGCGGTGAAATGCGTAGAGATCTGGAGGAATACCGGTGGCGAAGGCGGCCCCCTGGACA

AAGACTGACGCTCAGGTGCGAAAGCGTGGGGAGCAAACAGGATTAGATACCCTGGTAGTCCACGCCGTAA

ACGATGTCGATTTGGAGGCTGTGTCCTTGAGACGTGGCTTCCGGAGCTAACGCGTTAAATCGACCGCCTG

GGGAGTACGGCCGCAAGGTTAAAACTCAAATGAATTGACGGGGGCCCGCACAAGCGGTGGAGCATGTGGT

TTAATTCGATGCAACGCGAAGAACCTTACCTGGCCTTGACATGTCTGGAATCCTGTAGAGATACGGGAGT

GCCTTCGGGAATCAGAACACAGGTGCTGCATGGCTGTCGTCAGCTCGTGTCGTGAGATGTTGGGTTAAGT

CCCGCAACGAGCGCAACCCCTGTCCTTTGTTGCCAGCACGTAATGGTGGGAACTCAAGGGAGACTGCCGG

TGATAAACCGGAGGAAGGTGGGGATGACGTCAAGTCATCATGGCCCTTACGGCCAGGGCTACACACGTGC

TACAATGGCGCGTACAGAGGGCTGCAAGCTAGCGATAGTGAGCGAATCCCAAAAAGCGCGTCGTAGTCCG

GATCGGAGTCTGCAACTCGACTCCGTGAAGTCGGAATCGCTAGTAATCGCAAATCAGAATGTTGCGGTGA

ATACGTTCCCGGGCCTTGTACACACCGCCCGTCACACCATGGGAGTGGGTTGCACCAGAAGTAGATAGC

**MT380477**

CGGCATGAGGCAGCTACACATGCAGTCGAGCGGCAGCGGGAAAGTTAGCTTGCTTCTTTTGCCGGCGAGC

GGCGGACGGGTGAGTAATGCCTGGGGATCTGCCCAGTCGAGGGGGATAACTACTGGAAACGGTAGCTAAT

ACCGCATACGCCCTACGGGGGAAAGCAGGGGACCTTCGGGCCTTGCGCGATTGGATGAACCCAGGTGGGA

TTAGCTAGTTGGTGAGGTAATGGCTCACCAAGGCGACGATCCCTAGCTGGTCTGAGAGGATGATCAGCCA

CACTGGAACTGAGACACGGTCCAGACTCCTACGGGAGGCAGCAGTGGGGAATATTGCACAATGGGGGAAA

CCCTGATGCAGCCATGCCGCGTGTGTGAAGAAGGCCTTCGGGTTGTAAAGCACTTTCAGCGAGGAGGAAA

GGTTGGTAGCTAATAACTGCCAGCTGTGACGTTACTCGCAGAAGAAGCACCGGCTAACTCCGTGCCAGCA

GCCGCGGTAATACGGAGGGTGCAAGCGTTAATCGGAATTACTGGGCGTAAAGCGCACGCAGGCGGTTGGA

TAAGTTAGATGTGAAAGCCCCGGGCTCAACCTGGGAATTGCATTTAAAACTGTCCAGCTAGAGTCTTGGA

GAGGGGGGTAGAATTCCAGGTGTAGCGGTGAAATGCGTAGAGATCTGGAGGAATACCGGTGGCGAAGGCG

GCCCCCTGGACAAAGACTGACGCTCAGGTGCGAAAGCGTGGGGAGCAAACAGGATTAGCTACCCTGGTAG

TCCACGCCGTAAACGATGTCGATTTGGAGGCTGTGTCCTTGAGACGTGGCTTCCGGAGCTAACGCGTTAA

ATCGACCGCCTGGGGAGTACGGCCGCAAGGTTAAAACTCAAATGAATTGACGGGGGCCCGCACAAGCGGT

GGAGCATGTGGTTTAATTCGATGCAACGCGAAGAACCTTACCTGGCCTTGACATGTCTGGAATCCTGCAG

AGATGCGGGAGTGCCTTCGGGAATCAGAACACAGGTGCTGCATGGCTGTCGTCAGCTCGTGTCGTGAGAT

GTTGGGTTAAGTCCCGCAACGAGCGCAACCCCTGTCCTTTGTTGCCAGCACGTAATGGTGGGAACTCAAG

GGAGACTGCCGGTGATAAACCGGAGGAAGGTGGGGATGACGTCAAGTCATCATGGCCCTTACGGCCAGGG

CTACACACGTGCTACAATGGCGCGTACAGAGGGCTGCAAGCTAGCGATAGTGAGCGAATCCCAAAAAGCG

CGTCGTAGTCCGGATCGGAGTCTGCAACTCGACTCCGTGAAGTCGGAATCGCTAGTAATCGCAAATCAGA

ATGTTGCGGTGAATACGTTCCCGGGCCTTGTACACACCGCCCGTCACACCATGGGAGTGGGTTGCACCAG

AAAGTAGATAAGCTTAACCTTCGGGAGGGCGTTACCATCGATGA

**MT383121**

AGAGTTTGATCATGGCTCAGATTGAACGCTGGCGGCAGGCCTAACACATGCAAGTCGAGCGGCAGCGGGA

AAGTAGCTTGCTACTTTTGCCGGCGAGCGGCGGACGGGTGAGTAATGCCTGGGGATCTGCCCAGTCGAGG

GGGATAACTACTGGAAACGGTAGCTAATACCGCATACGCCCTACGGGGGAAAGCAGGGGACCTTCGGGCC

TTGCGCGATTGGATGAACCCAGGTGGGATTAGCTAGTTGGTGAGGTAATGGCTCACCAAGGCGACGATCC

CTAGCTGGTCTGAGAGGATGATCAGCCACACTGGAACTGAGACACGGTCCAGACTCCTACGGGAGGCAGC

AGTGGGGAATATTGCACAATGGGGGAAACCCTGATGCAGCCATGCCGCGTGTGTGAAGAAGGCCTTCGGG

TTGTAAAGCACTTTCAGCGAGGAGGAAAGGTTGGTAGCTAATAACTGCCAGCTGTGACGTTACTCGCAGA

AGAAGCACCGGCTAACTCCGTGCCAGCAGCCGCGGTAATACGGAGGGTGCAAGCGTTAATCGGAATTACT

GGGCGTAAAGCGCACGCAGGCGGTTGGATAAGTTAGATGTGAAAGCCCCGGGCTCAACCTGGGAATTGCA

TTTAAAACTGTCCAGCTAGAGTCTTGTAGAGGGGGGTAGAATTCCAGGTGTAGCGGTGAAATGCGTAGAG

ATCTGGAGGAATACCGGTGGCGAAGGCGGCCCCCTGGACAAAGACTGACGCTCAGGTGCGAAAGCGTGGG

GAGCAAACAGGATTAGATACCCTGGTAGTCCACGCCGTAAACGATGTCGATTTGGAGGCTGTGTCCTTGA

GACGTGGCTTCCGGAGCTAACGCGTTAAATCGACCGCCTGGGGAGTACGGCCGCAAGGTTAAAACTCAAA

TGAATTGACGGGGGCCCGCACAAGCGGTGGAGCATGTGGTTTAATTCGATGCAACGCGAAGAACCTTACC

TGGCCTTGACATGTCTGGAATCCTGCAGAGATGCGGGAGTGCCTTCGGGAATCAGAACACAGGTGCTGCA

TGGCTGTCGTCAGCTCGTGTCGTGAGATGTTGGGTTAAGTCCCGCAACGAGCGCAACCCCTGTCCTTTGT

TGCCAGCACGTAATGGTGGGAACTCAAGGGAGACTGCCGGTGGTATACCGGAGGAAGGTGGGGATGACGT

CAAGTCATCATGGCCCTTACGGCCAGGGCTACACACGTGCTACAATGGCGCGTACAGAGGGCTGCAAGCT

AGCGATAGTGAGCGAATCCCAAAAAGCGCGTCGTAGTCCGGATCGGAGTCTGCAACTCGACTCCGTGAAG

TCGGAATCGCTAGTAATCGCAAATCAGAATGTTGCGGTGAATACGTTCCCGGGCCTTGTACACACCGCCC

GTCACACCATGGGAGTGGGTTGCACCAGAAGTAGATAGCTTAACCTTCGGGAGGGCGTTTACCACGGTGT

GATTCATGACTGGGGTGAAGTCGTAACAAGGTACC

**MT383124**

AGAGTTTGATCATGGCTCAGATTGAACGCTGGCGGCAGGCCTAACACATGCAAGTCGAGCGGCAGCGGGA

AAGTAGCTTGCTACTTTTGCCGGCGAGCGGCGGACGGGTGAGTAATGCCTGGGGATCTGCCCAGTCGAGG

GGGATAACTACTGGAAACGGTAGCTAATACCGCATACGCCCTACGGGGGAAAGCAGGGGACCTTCGGGCC

TTGCGCGATTGGATGAACCCAGGTGGGATTAGCTAGTTGGTGAGGTAATGGCTCACCAAGGCGACGATCC

CTAGCTGGTCTGAGAGGATGATCAGCCACACTGGAACTGAGACACGGTCCAGACTCCTACGGGAGGCAGC

AGTGGGGAATATTGCACAATGGGGGAAACCCTGATGCAGCCATGCCGCGTGTGTGAAGAAGGCCTTCGGG

TTGTAAAGCACTTTCAGCGAGGAGGAAAGGTTGGTAGCTAATAACTGCCAGCTGTGACGTTACTCGCAGA

AGAAGCACCGGCTAACTCCGTGCCAGCAGCCGCGGTAATACGGAGGGTGCAAGCGTTAATCGGAATTACT

GGGCGTAAAGCGCACGCAGGCGGTTGGATAAGTTAGATGTGAAAGCCCCGGGCTCAACCTGGGAATTGCA

TTTAAAACTGTCCAGCTAGAGTCTTGTAGAGGGGGGTATAATTCCAGGTGTAGCGGTGAAATGCGTATAG

ATCTGGAGGAATACCGGTGGCGAAAGCGGCCCCCTGGACAAAGACTGACGCTCAGGTGCGAAAGCGTGGG

GAGCAAACAGGATTAGATACCCTGGTAGTCCACGCCGTAAACGATGTCGATTTGGAGGCTGTGTCGTTGA

GACATGCCTTCTGGAGCTAACGCGTTAAATCGACCGCCTGGGGAGTACGGCCGCAAGGTTAAAACTCAAA

TGAATTGACGGGGGCCCGCACAAGCGGTGGAGCATGTGGTTTAATTCAATGCAACGCGAAGAACCTTACC

TGGCCTTGACATGTCTGGAATCCTGCAGAGATGCGGGAGTGCCTTCGGGAATCAGAACACAGGAGCTGCA

TGGCTGTCGTCAGCTCGTGTCGTGAGATGTTGGGTTAAGTCCCGCAACGAGCGCAACCCCTGTCCTTTGT

TGCCAGCACGTAATGGTGGGAACTCAAGGGAGACTGCCGGTGATAAACCGGAGGAAGGTGGGGATGACGT

CAAGTCATCATGGCCCTTACGGCCAGGGCTACACACGTGCTACAATGGCGCGTACAGAGGGCTGCAAGCT

AGCGATAGTGAGCGAATCCCAAAAAGCGCGTCGTAGTCCGGATCGGAGTCTGCAACTCGACTCCGTGAAG

TCGGAATCGCTAGTAATCGCAAATCAGAATGTTGCGGTGAATACGTTCCCGGGCCTTGTACACACCGCCC

GTCACACCATGGGAGTGGGTTGCACCAGAAGTAGATAGCTTAACCTTCGGGAGGGCGTTTACCACGGTGT

GATTCATGACTGGGGTGAAGTCGTAACAAGGTACC

**MT384337**

GACGCTGGCGGCGGCCTACACATGCAAGTCGAGCGGCAGCGGGAAAGTAGCTTGCTACTTTTGCCGGCGA

GCGGCGGACGGGTGAGTAATGCCTGGGGATCTGCCCAGTCGAGGGGGATAACTACTGGAAACGGTAGCTA

ATACCGCATACGCCCTACGGGGGAAAGCAGGGGACCTTCGGGCCTTGCGCGATTGGATGAACCCAGGTGG

GATTAGCTAGTTGGTGAGGTAATGGCTCACCAAGGCGACGATCCCTAGCTGGTCTGAGAGGATGATCAGC

CACACTGGAACTGAGACACGGTCCAGACTCCTACGGGAGGCAGCAGTGGGGAATATTGCACAATGGGGGA

AACCCTGATGCAGCCATGCCGCGTGTGTGAAGAAGGCCTTCGGGTTGTAAAGCACTTTCAGCGAGGAGGA

AAGGTTGGTAGCTAATAACTGCCAGCTGTGACGTTACTCGCAGAAGAAGCACCGGCTAACTCCGTGCCAG

CAGCCGCGGTAATACGGAGGGTGCAAGCGTTAATCGGAATTACTGGGCGTAAAGCGCACGCAGGCGGTTG

GATAAGTTAGATGTGAAAGCCCCGGGCTCAACCTGGGAATTGCATTTAAAACTGTCCAGCTAGAGTCTTG

TAGAGGGGGGTAGAATTCCAGGTGTAGCGGTGAAATGCGTAGAGATCTGGAGGAATACCGGTGGCGAAGG

CGGCCCCCTGGACAAAGACTGACGCTCAGGTGCGAAAGCGTGGGGAGCAAACAGGATTAGATACCCTGGT

AGTCCACGCCGTAAACGATGTCGATTTGGAGGCTGGGTCCTTGAGACGTGGCTTCCGGAGCTAACGCGTT

AAATCGACCGCCTGGGGAGTACGGCCGCAAGGTTAAAACTCAAATGAATTGACGGGGGCCCGCACAAGCG

GTGGAGCATGTGGTTTAATTCGATGCAACGCGAAGAACCTTACCTGGCCTTGACATGTCTGGAATCCTGT

AGAGATACGGGAGTGCCTTCGGGAATCAGAACACAGGTGCTGCATGGCTGTCGTCAGCTCGTGTCGTGAG

ATGTTGGGTTAAGTCCCGCAACGAGCGCAACCCCTGTCCTTTGTTGCCAGCACGTAATGGTGGGAACTCA

AGGGAGACTGCCGGTGATAAACCGGAGGAAGGTGGGGATGACGTCAAGTCATCATGGCCCTTACGGCCAG

GGCTACACACGTGCTACAATGGCGCGTACAGAGGGCTGCAAGCTAGCGATAGTGAGCGAATCCCAAAAAG

CGCGTCGTAGTCCGGATCGGAGTCTGCAACTCGACTCCGTGAAGTCGGAATCGCTAGTAATCGCAAATCA

GAATGTTGCGGTGAATACGTTCCCGGGCCTTGTACACACCGCCCGTCACACCATGGGAGTGGGTTGCACC

AGAAGTAGATAGCTTAACCTTCGGGAGGGCGTTTACCACGG

**MT396085**

CTACACATGCAAGTCGAGCGGCAGCGGGCAAGTAGCTTGCTACTTTTGCCGGCGAGCGGCGGACGGGTGA

GTAATGCCTGGGGATCTGCCCAGTCGAGGGGGATAACTACTGGAAACGGTAGCTAATACCGCATACGCCC

TACGGGGGAAAGCAGGGGACCTTCGGGCCTTGCGCGATTGGATGAACCCAGGTGGGATTAGCTAGTTGGT

GAGGTAATGGCTCACCAAGGCGACGATCCCTAGCTGGTCTGAGAGGATGATCAGCCACACTGGAACTGAG

ACACGGTCCAGACTCCTACGGGAGGCAGCAGTGGGGAATATTGCACAATGGGGGAAACCCTGATGCAGCC

ATGCCGCGTGTGTGAAGAAGGCCTTCGGGTTGTAAAGCACTTTCAGCGAGGAGGAAAGGTTGGTAGCTAA

TAACTGCCAGCTGTGACGTTACTCGCAGAAGAAGCACCGGCTAACTCCGTGCCAGCAGCCGCGGTAATAC

GGAGGGTGCAAGCGTTAATCGGAATTACTGGGCGTAAAGCGCACGCAGGCGGTTGGATAAGTTAGATGTG

AAAGCCCCGGGCTCAACCTGGGAATTGCATGTAGAACTGTCCAGCTAGAGTCTTGTAGAGGGGGGTAGAA

TTCCAGGTGTAGCGGTGAAATGCGTAGAGATCTGGAGGAATACCGGGGGGGAAAGCGGCCCCTGGACAAA

GACTGACGCTCAGGTGCGAAAAGCGTGGGGAGCAAACGGGATTAGATACCCTGGTAGTCCACGCCGGTAA

ACCATCGTCGATTTGGAGGCTGTTTCTTTGAAACGTGGCTTTCGGAAGCTAACTCCTTAAAA

**MT384421**

CTACACATGCAAGTCGAGCGGCAGCGGGCAAGTAGCTTGCTACTTTTGCCGGCGAGCGGCGGACGGGTGA

GTAATGCCTGGGGATCTGCCCAGTCGAGGGGGATAACTACTGGAAACGGTAGCTAATACCGCATACGCCC

TACGGGGGAAAGCAGGGGACCTTCGGGCCTTGCGCGATTGGATGAACCCAGGTGGGATTAGCTAGTTGGT

GAGGTAATGGCTCACCAAGGCGACGATCCCTAGCTGGTCTGAGAGGATGATCAGCCACACTGGAACTGAG

ACACGGTCCAGACTCCTACGGGAGGCAGCAGTGGGGAATATTGCACAATGGGGGAAACCCTGATGCAGCC

ATGCCGCGTGTGTGAAGAAGGCCTTCGGGTTGTAAAGCACTTTCAGCGAGGAGGAAAGGTTGGTAGCTAA

TAACTGCCAGCTGTGACGTTACTCGCAGAAGAAGCACCGGCTAACTCCGTGCCAGCAGCCGCGGTAATAC

GGAGGGTGCAAGCGTTAATCGGAATTACTGGGCGTAAAGCGCACGCAGGCGGTTGGATAAGTTAGATGTG

AAAGCCCCGGGCTCAACCTGGGAATTGCATGTAGAACTGTCCAGCTAGAGTCTTGTAGAGGGGGGTAGAA

TTCCAGGTGTAGCGGTGAAATGCGTAGAGATCTGGAGGAATACCGGGGGGGAAAGCGGCCCCTGGACAAA

GACTGACGCTCAGGTGCGAAAAGCGTGGGGAGCAAACGGGATTAGATACCCTGGTAGTCCACGCCGGTAA

ACCATCGTCGATTTGGAGGCTGTTTCTTTGAAACGTGGCTTTCGGAAGCTAACTCCTTAAAA

**MT385097**

GCCTACCATGCAAGTCGAGCGGCAGCGGGAAAGTAGCTTGCTACTTTTGCCGGCGAGCGGCGGACGGGTG

AGTAATGCCTGGGGATCTGCCCAGTCGAGGGGGATAACTACTGGAAACGGTAGCTAATACCGCATACGCC

CTACGGGGGAAAGCAGGGGACCTTCGGGCCTTGCGCGATTGGATGAACCCAGGTGGGATTAGCTAGTTGG

TGAGGTAATGGCTCACCAAGGCGACGATCCCTAGCTGGTCTGAGAGGATGATCAGCCACACTGGAACTGA

GACACGGTCCAGACTCCTACGGGAGGCAGCAGTGGGGAATATTGCACAATGGGGGAAACCCTGATGCAGC

CATGCCGCGTGTGTGAAGAAGGCCTTCGGGTTGTAAAGCACTTTCAGCGAGGAGGAAAGGTTGGTAGCTA

ATAACTGCCAGCTGTGACGTTACTCGGAGAACAAGCACCGGTTAACTCCGTGCCACCTGCCGCGGTAATA

CGGAGGGTGCAAGCGTTAATCGAATTTACTGGGCGTAAAGCGCACGCAGGCGGTTGGATAAGTTAGATGT

GAAATCCCCGGGCTCAACCTGGGAATTGCATTTAAAACTGTCCAGCTAGAGTCTTGTAGAGGGGGGTAGA

ATTCCAGGTGTAGCGGTGAAATGCGTAGAGATCTGGAGGAATACCGGTGGCGAAGGCGGCCCCCTGGACA

AAGACTGACGCTCAGGTGAGAAAGCGTGGGGAGCAAACAGGATTAGATACCGTGGTAGTCCACGCCGTAA

ACGATGTCGATTTGGAGGCTGTGTCCTTGAGACGTGGTTTCCGGAGCTAACGCGTTAAATTGACCGCCTG

GGGAGTACGGCCGCAAGGTTAAAACTCAAATGAATTGACGGGGGCCCGCACAAGCGGTGGAGCATGTGGT

TTAATTCGATGCAACGCGAAGAACCTTACCTGGCCTTGACATGTCTGGAATCCTGTAGAGATACGGGAGT

GCCTTCGGGAATCAGAACACAGGTGCTGCATGGCTGTCGTCAGCTCGTGTCGTGAGATGTTGGGTTAAGT

CCCGCAACGAGCGCAACCCCTGTCCTTTGTTGCCAGCACGTAATGGTGGGAACTCAAGGGAGACTGCCGG

TGATAAACCGGAGGAAGGTGGGGATGACGTCAAGTCATCATGGCCCTTACGGCCAGGGCTACACACGTGC

TACAATGGCGCGTACAGAGGGCTGCAAGCTAGCGATAGTGAGCGAATCCCAAAAAGCGCGTCGTAGTCCG

GATCGGAGTCTGCAACTCGACTCCGTGAAGTCGGAATCGCTAGTAATCGCAAATCAGAATGTTGCGGTGA

ATACGT

**MT393937**

GCGGGAAAGTAGCTTGCTACTTTTGCCGGCGAGCGGCGGACGGGTGAGTAATGCCTGGGGATCTGCCCAG

TCTAGGGGGATAACTACTGGAAACGGTAGCTAATACCGCATACGCCCTACGGGGGAAAGCAGGGGACCTT

CGGGCCTTGCGCGATTGGATGAACCCAGGTGGGATTAGCTAGTTGGTGAGGTAACGGCTCACCAAGGCGA

CGATCCCTAGCTGGTCTGAGAGGATGATCAGCCACACTGGAACTGAGACACGGTCCACACTCCTACGGGA

GGCAGCAGTGGGGAATATTGCACAATGGGGGAAACCCTGATGCAGCCATGCCGCGTGTGTGAAGAAGGCC

TTCGGGTTGTAAAGCACTTTCAGCGAGGAGGAAAGGTCAGTAGCTAATATCTGCTGGCTGTGACGTTACT

CGCAGAAGAAGCACCGGCTAACTCCGTGCCAGCAGCCGCGGTAATACGGAGGGTGCAAGCGTTAATCGGA

ATTACTGGGCGTAAAGCGCACGCAGGCGGTTGGATAAGTTAGATGTGAAAGCCCCGGGCTCAACCTGGGA

ATTGCATTTAAAACTGTCCAGCTAGAGTCTTGTAGAGGGGGGTAGAATTCCAGGTGTAGCGGTGAAATGC

GTAGAGATCTGGAGGAATACCGGTGGCGAAGGCGGCCTCCTGGACACAGACTGACGCTCAGGTGCGAAAG

CGTGGGGAGCAAACAGGATTAGATACCCTGGTAGTCCACGCCGTACACGATGTCGATTTGGAGGCTGTGT

CCTTGAGACGTGGCTTCCGGAGCTAACGCGTTAGATCGACCGCCTGGGGAGTACGGCCGCAAGGTTAAAA

CTCAGATGAATTGACGGGGGCCCGCACAAGCGGTGGAGCATGTGGTTTATTCGATGCAA

**MT393933**

GATTGACGCTGCGGCAGGCCTAACACATGCAAGTCGAGCGGCAGCGGGAAAGTAGCTTGCTACTTTTGCC

GGCGAGCGGCGGACGGGTGAGTAATGCCTGGGAAATTGCCCAGTCGAGGGGGATAACAGTTGGAAACGAC

TGCTAATACCGCATACGCCCTACGGGGGAAAGCAGGGGACCTTCGGGCCTTGCGCGATTGGATATGCCCA

GGTGGGATTAGCTAGTTGGTGAGGTAATGGCTCACCAAGGCGACGATCCCTAGCTGGTCTGAGAGGATGA

TCAGCCACACTGGAACTGAGACACGGTCCAGACTCCTACGGGAGGCAGCAGTGGGGAATATTGCACAATG

GGGGAAACCCTGATGCAGCCATGCCGCGTGTGTGAAGAAGGCCTTCGGGTTGTAAAGCACTTTCAGCGAG

GAGGAAAGGTCAGTAGCTAATATCTGCTGACTGTGACGTTACTCGCAGAAGAAGCACCGGCTAACTCCGT

GCCAGCAGCCGCGGTAATACGGAGGGTGCAAGCGTTAATCGGAATTACTGGGCGTAAAGCGCACGCAGGC

GGTTGGATAAGTTAGATGTGAAAGCCCCGGGCTCAACCTGGGAATTGCATTTAAAACTGTCCAGCTAGAG

TCTTGTAGAGGGGGGTAGAATTCCAGGTGTAGCGGTGAAATGCGTAGAGATCTGGAGGAATACCGGTGGC

GAAGGCGGCCCCCTGGACAAAGACTGACGCTCAGGTGCGAAAGCGTGGGGAGCAAACAGGATTAGATACC

CTGGTAGTCCACGCCGTAAACGATGTCGATTTGGAGGCTGTGTCCTTGAGACGTGGCTTCCGGAACTAAC

GCGTTAAATCGACCGCCTGGGGAGTACGGCCGCAAGGTTAGAACTCAAATGAATTGACGGGGGCCCGCAC

CAGCGGGGGAGCATGTGGTTTAATTCCATGCACCCGAAGAACCTTACCTGGCCTTGACATGTCTGGAATC

CTGCAGAGATGCGGGAGTGCCTTCGGGAATCAGAACACAGGTGCTGCATGGCTGTCGTCAGCTCGTGTCG

TGAGATGTTGGGTTAAGTCCCGCAACGAGCGCAACCCCTGTCCTTTGTTGCCAGCACGTAATGGTGGGAA

CTCAAGGGAGACTGCCGGTGATAAACCGGAGGAAGGTGGGGATGACGTCAAGTCATCATGGCCCTTACGG

CCAGGGCTACACACGTGCTACAATGGCGCGTACAGAGGGCTGCAAGCTAGCGATAGTGAGCGAATCCCAA

AAAGCGCGTCGTAGTCCGGATTGGAGTCTGCAACTCGACTCCATGAAGTCGGAATCGCTAGTAATCGCAA

ATCAGAATGTTGCGGTGAATACGTTCCCGGGCCTTGTACACACCGCCCGTCACACCATGGGAGTGGGTTG

CACCAGAAGTAGATAGCTTAACCTTCGGGAGGGCGTTTACCACGGTGTGATTCATGACTGGGGTGAGTCA

CAAAGGGGAAACCCCCCAAAACCCTACCCCCTAAATTTTTCGCCTCAAGGTTTCCAACTTTTTTCCCCCT

CCACCAGGCACTTATCCCAGACTTTCCTCACCCGTCCGCCACTCTTCACCCTAGAGCAC

**MT393941**

GCAAGTCGAGCGGCAGCGGGAAAGTAGCTTGCTACTTTTGCCGGCGAGCGGCGGACGGGTGAGTAATGCC

TGGGAAATTGCCCAGTCGAGGGGGATAACAGTTGGAAACGACTGCTAATACCGCATACGCCCTACGGGGG

AAAGCAGGGGACCTTCGGGCCTTGCGCGATTGGATATGCCCAGGTGGGATTAGCTAGTTGGTGAGGTAAT

GGCTCACCAAGGCGACGATCCCTAGCTGGTCTGAGAGGATGATCAGCCACACTGGAACTGAGACACGGTC

CAGACTCCTACGGGAGGCAGCAGTGGGGAATATTGCACAATGGGGGAAACCCTGATGCAGCCATGCCGCG

TGTGTGAAGAAGGCCTTCGGGTTGTAAAGCACTTTCAGCGAGGAGGAAAGGTCAGTAGCTAATATCTGCT

GACTGTGACGTTACTCGCAGAAGAAGCACCGGCTAACTCCGTGCCAGCAGCCGCGGTAATACGGAGGGTG

CAAGCGTTAATCGGAATTACTGGGCGTAAAGCGCACGCAGGCGGTTGGATAAGTTAGATGTGAAAGCCCC

GGGCTCAACCTGGGAATTGCATTTAAAACTGTCCAGCTAGAGTCTTGTAGAGGGGGGTAGAATTCCAGGT

GTAGCGGTGAAATGCGTAGAGATCTGGAGGAATACCGGTGGCGAAGGCGGCCCCCTGGACAAAGACTGAC

GCTCAGGTGCGAAAGCGTGGGGAGCAAACAGGATTAGATACCCTGGTAGTCCACGCCGTAAACGATGTCG

ATTTGGAGGCTGTGTCCTTGAGACGTGGCTTCCGGAGCTAACGCGTTAAATCGACCGCCTGGGGAGTACG

GCCGCAAGGTTAAACTCAAATGAATTGACGGGGGCCCGCACAAGCGGTGGAGCATGTGGTTAATTCGATG

CACGCGAGAACCTTACTGGCCTTG

**MT393944**

TGCAAGTCGAGCGGCAGCGGGAAAGTAGCTTGCTACTTTTGCCGGCGAGCGGCGGACGGGTGAGTAATGC

CTGGGAAATTGCCCAGTCGAGGGGGATAACAGTTGGAAACGACTGCTAATACCGCATACGCCCTACGGGG

GAAAGCAGGGGACCTTCGGGCCTTGCGCGATTGGATATGCCCAGGTGGGATTAGCTAGTTGGTGAGGTAA

TGGCTCACCAAGGCGACGATCCCTAGCTGGTCTGAGAGGATGATCAGCCACACTGGAACTGAGACACGGT

CCAGACTCCTACGGGAGGCAGCAGTGGGGAATATTGCACAATGGGGGAAACCCTGATGCAGCCATGCCGC

GTGTGTGAAGAAGGCCTTCGGGTTGTAAAGCACTTTCAGCGAGGAGGAAAGGTCAGTAGCTAATATCTGC

TGACTGTGACGTTACTCGCAGAAGAAGCACCGGCTAACTCCGTGCCAGCAGCCGCGGTAATACGGAGGGT

GCAAGCGTTAATCGGAATTACTGGGCGTAAAGCGCACGCAGGCGGTTGGATAAGTTAGATGTGAAAGCCC

CGGGCTCAACCTGGGAATTGCATTTAAAACTGTCCAGCTAGAGTCTTGTAGAGGGGGGTAGAATTCCAGG

TGTAGCGGTGAAATGCGTAGAGATCTGGAGGAATACCGGTGGCGAAGGCGGCCCCCTGGACAAAGACTGA

CGCTCAGGTGCGAAAGCGTGGGGAGCAAACAGGATTAGATACCCTGGTAGTCCACGCCGTAAACGATGTC

GATTTGGAGGCTGTGTCCTTGAGACGTGGCTTCCGGAGCTAACGCGTTAAATCGACCGCCTGGGGAGTAC

GGCCCAAGGGTAAAACTCAAATGAATTGACGGGGGCC

**MT393945**

GCGGCAGCGGGAAAGTAGCTTGCTACTTTTGCCGGCGAGCGGCGGACGGGTGAGTAATGCCTGGGAAATT

GCCCAGTCGAGGGGGATAACAGTTGGAAACGACTGCTAATACCGCATACGCCCTACGGGGGAAAGCAGGG

GACCTTCGGGCCTTGCGCGATTGGATATGCCCAGGTGGGATTAGCTAGTTGGTGAGGTAATGGCTCACCA

AGGCGACGATCCCTAGCTGGTCTGAGAGGATGATCAGCCACACTGGAACTGAGACACGGTCCAGACTCCT

ACGGGAGGCAGCAGTGGGGAATATTGCACAATGGGGGAAACCCTGATGCAGCCATGCCGCGTGTGTGAAG

AAGGCCTTCGGGTTGTAAAGCACTTTCAGCGAGGAGGAAAGGTCAGTAGCTAATATCTGCTGACTGTGAC

GTTACTCGCAGAAGAAGCACCGGCTAACTCCGTGCCAGCAGCCGCGGTAATACGGAGGGTGCAAGCGTTA

ATCGGAATTACTGGGCGTAAAGCGCACGCAGGCGGTTGGATAAGTTAGATGTGAAAGCCCCGGGCTCAAC

CTGGGAATTGCATTTAAAACTGTCCAGCTAGAGTCTTGTAGAGGGGGGTAGAATTCCAGGTGTAGCGGTG

AAATGCGTAGAGATCTGGAGGAATACCGGTGGCGAAGGCGGCCCCCTGGACAAAGACTGACGCTCAGGTG

CGAAAGCGTGGGGAGCAAACAGGATTAGATACCCTGGTAGTCCACGCCGTAAACGATGTCGATTTGGAGG

CTGTGTCCTTGAGACGTGGCTTCCGGAGCTAACGCGTTAAATCGACCGCCTGGGGAGTACGGCCGCAAGG

TTAAAACTCAAATGAATTGACGGGGGCCCGCACAAGCGGTGGAGCATGTGGTTAATT

**MT395673**

TGTCGCAGCGAAGCACAAGCAGCCAGGCGAGAGGAGGGGGAAAGAGGGGCGAGTTAGGGGAAGTGGCGGA

GGGGCGAAAAAGTTGGGGAAGTGGCAGGCGAGGGGGATAACAGTTGGAAACGAGTGCTAATACCGCATAC

GCCCTACGGGGGAAAGCAGGGGACCTTCGGGCCTTGCGCGATTGGATATGCCCAGGTGGGATTAGCTAGT

TGGTGAGGTAATGGCTCACCAAGGCGACGATCCCTAGCTGGTCTGAGAGGATGATCAGCCACACTGGAAC

TGAGACACGGTCCAGACTCCTACGGGAGGCAGCAGTGGGGAATATTGCACAATGGGGGAAACCCTGATGC

AGCCATGCCGCGTGTGTGAAGAAGGCCTTCGGGTTGTAAAGCACTTTCAGCGAGGAGGAAAGGTTGTGGC

CTAATACGTATCAACTGTGACGTTACTCGCAGAAGAAGCACCGGCTAACTCCGTGCCAGCAGCCGCGGTA

ATACGGAGGGTGCAAGCGTTAATCGGAATTACTGGGCGTAAAGCGCACGCAGGCGGTTGGATAAGTTAGA

TGTGAAAGCCCCGGGCTCAACCTGGGAATTGCATTTAAAACTGTCCAGCTAGAGTCTTGTAGAGGGGGGT

AGAATTCCAGGTGTAGCGGTGAAATGCGTAGAGATCTGGAGGAATACCGGTGGCGAAGGCGGCCCCCTGG

ACAAAGACTGACGCTCAGGTGCGAAAGCGTGGGGAGCAGACAGGATTAGATACCCTGGTAGTCCACGCCG

TAAACGATGTCGATTTGGAGGCTGTGTCCTTGAGACGTGGCTTCCGGAGCTAACGCGTTAAATCGACCGC

CTGGGGAGTACGGCCGCAAGGTTAAGACTCAAATGAATTGACGGGGGCCCGCACAAGCGGTGGAGCATGT

GGTTTAATTCGATGCAACGCGGAGAACCTTACCTGGCCTTGACATGTCTGGAATCCTGCAAAGATGCGGG

AGTGCCTTCGGGAATCAGAAC

**MT396230**

TGGGGGGGGGGGCGGCCTACACATGCAAGTCGAGCGGCAGCGGGAAAGTAGCTTGCTACTTTTGCCGGCG

AGCGGCGGACGGGTGAGTAATGCCTGGGGATCTGCCCAGTCGAGGGGGATAACTACTGGAAACGGTAGCT

AATACCGCATACGCCCTACGGGGGAAAGCAGGGGACCTTCGGGCCTTGCGCGATTGGATGAACCCAGGTG

GGATTAGCTAGTTGGTGAGGTAATGGCTCACCAAGGCGACGATCCCTAGCTGGTCTGAGAGGATGATCAG

CCACACTGGAACTGAGACACGGTCCAGACTCCTACGGGAGGCAGCAGTGGGGAATATTGCACAATGGGGG

AAACCCTGATGCAGCCATGCCGCGTGTGTGAAGAAGGCCTTCGGGTTGTAAAGCACTTTCAGCGAGGAGG

AAAGGTTGGTAGCTAATAACTGCCAGCTGTGACGTTACTCGCAGAAGAAGCACCGGCTAACTCCGTGCCA

GCAGCCGCGGTAATACGGAGGGTGCAAGCGTTAATCGGAATTACTGGGCGTAAAGCGCACGCAGGCGGTT

GGATAAGTTAGATGTGAAAGCCCCGGGCTCAACCTGGGAATTGCATTTAAAACTGTCCAGCTAGAGTCTT

GTAGAGGGGGGTAGAATTCCAGGTGTAGCGGTGAAATGCGTAGAGATCTGGAGGAATACCGGTGGCGAAG

GCGGCCCCCTGGACAAAGACTGACGCTCAGGTGCGAAAGCGTGGTGAGCAGACAGGATTATATACCCTGG

TAGTCCACGCCGTAAACGATGTCGATTTGGAGGCTGTGTCCTTGATACGTGCCTTCCGGAGCTAACGCGT

TAAATCGACCGCCTGGGGAGTACGGCCGCAGGGTTAAAACTCAAATGAATTGACGGGGGCCCGCACAAGC

GGTGGAGCATGTGGGTTTAATTCGATGCAACGCGAAGAACCTTACCTGGCCTTGACATGTCTGGAATCCT

GTAGAGATACGGGAGTGCCTTCGGGAATCAGAACACAGGTGCTGCATGGCTGTCGTCAGCTCGTGTCGTG

AGATGTTGGGTTAAGTCCCGCAACGAGCGCAACCCCTGTCCTTTGTTGCCAGCACGTAATGGTGGGAACT

CAAGGGAGACTGCCGGTGATAAACCGGAGGAAGGTGGGGATGACGTCAAGTCATCATGGCCCTTACGGCC

AGGGCTACACACGTGCTACAATGGCGCGTACAGAGGGCTGCAAGCTAGCGATAGTGAGCGAATCCCAAAA

AGCGCGTCGTAGTCCGGATCGGAGTCTGCAACTCGACTCCGTGAAGTCGGAATCGCTAGTAATCGCAAAT

CAGAATGTTGCGGTGAATACGTTCCCGGGCCTTGTACACACCGCCCGTCACACCATGGGAGTGGGTTGCA

CCAGAAGTAGATAGCTTAACCTTCGGGAGGGCGTTACCACGGTTATTCATGCGGG

**MT396436**

AGCTACCATGCAAGTCGAGCGGCAGCGGGAAAGTAGCTTGCTACTTTTGCCGGCGAGCGGCGGACGGGTG

AGTAATGCCTGGGAAATTGCCCAGTCGAGGGGGATAACAGTTGGAAACGACTGCTAATACCGCATACGCC

CTACGGGGGAAAGCAGGGGACCTTCGGGCCTTGCGCGATTGGATATGCCCAGGTGGGATTAGCTAGTTGG

TGAGGTAATGGCTCACCAAGGCGACGATCCCTAGCTGGTCTGAGAGGATGATCAGCCACACTGGAACTGA

GACACGGTCCAGACTCCTACGGGAGGCAGCAGTGGGGAATATTGCACAATGGGGGAAACCCTGATGCAGC

CATGCCGCGTGTGTGAAGAAGGCCTTCGGGTTGTAAAGCACTTTCAGCGAGGAGGAAAGGTTGATGCCTA

ATACGTATCAACTGTGACGTTACTCGCAGAAGAAGCACCGGCTAACTCCGTGCCAGCAGCCGCGGTAATA

CGGAGGGTGCAAGCGTTAATCGGAATTACTGGGCGTAAAGCGCACGCAGGCGGTTGGATAAGTTAGATGT

GAAAGCCCCGGGCTCAACCTGGGAATTGCATTTAAAACTGTCCAGCTAGAGTCTTGTAGAGGGGGGTAGA

ATTCCAGGTGTAGCGGTGAAATGCGTAGAGATCTGGAGGAATACCGGTGGCGAAGGCGGCCCCCTGGACA

AAGACTGACGCTCAGGTGCGAAAGCGTGGGGAGCAAACAGGATTAGATACCCTGGTAGTCCACGCCGTAA

ACGATGTCGATTTGGAGGCTGTGTCCTTGAGACGTGGCTTCCGGAGCTAACGCGTTAAATCGACCGCCTG

GGGAGTACGGCCGCAAGGTTAAAACTCAAATGAATTGACGGGGGCCCGCACGAGCGGTGGAGCATGTGGT

TTAATTCGATGCAACGCGAAGAACCTTACCTGGCCTTGACATGTCTGGAATCCTGCAGAGATGCGGGAGT

GCCTTCGGGAATCAGAACACAGGTGCTGCATGGCTGTCGTCAGCTCGTGTCGTGAGATGTTGGGTTAAGT

CCCGCAACGAGCGCAACCCCTGTCCTTTGTTGCCAGCACGTAATGGTGGGAACTCAAGGGAGACTGCCGG

TGATAAACCGGAGGAAGGTGGGGATGACGTCAAGTCATCATGGCCCTTACGGCCAGGGCTACACACGTGC

TACAATGGCGCGTACAGAGGGCTGCAAGCTAGCGATAGTGAGCGAATCCCAAAAAGCGCGTCGTAGTCCG

GATCGGAGTCTGCAACTCGACTCCGTGAAGTCGGAATCGCTAGTAATCGCAAATCAGAATGTTGCGGTGA

ATACGTTCCCGGGCCTTGTACACACCGCCCGTCACACCATGGGAGTGGGTTGCACCAGAAGTAGATAGCT

TAACCTTCGGGAGGGCGTTACACGGAGAATTAGGGGACTGGGGGTGAAGTCGTAACAAGGTAACCCTAGG

GGAACTGGTGTGTGATAACCTCCCCCTTATATATAAAGAGGGGTAGAGGT

**MT396445**

GGCCATGGGGGGCGGCCTACACATGCAAGTCGAGCGGCAGCGGGAAAGTAGCTTGCTACTTTTGCCGGCG

AGCGGCGGACGGGTGAGTAATGCCTGGGAAATTGCCCAGTCGAGGGGGATAACAGTTGGAAACGACTGCT

AATACCGCATACGCCCTACGGGGGAAAGCAGGGGACCTTCGGGCCTTGCGCGATTGGATATGCCCAGGTG

GGATTAGCTAGTTGGTGAGGTAATGGCTCACCAAGGCGACGATCCCTAGCTGGTCTGAGAGGATGATCAG

CCACACTGGAACTGAGACACGGTCCAGACTCCTACGGGAGGCAGCAGTGGGGAATATTGCACAATGGGGG

AAACCCTGATGCAGCCATGCCGCGTGTGTGAAGAAGGCCTTCGGGTTGTAAAGCACTTTCAGCGAGGAGG

AAAGGTTGATGCCTAATACGTATCAACTGTGACGTTACTCGCAGAAGAAGCACCGGCTAACTCCGTGCCA

GCAGCCGCGGTAATACGGAGGGTGCAAGCGTTAATCGGAATTACTGGGCGTAAAGCGCACGCAGGCGGTT

GGATAAGTTAGATGTGAAAGCCCCGGGCTCAACCTGGGAATTGCATTTAAAACTGTCCAGCTTGAGTCTT

GTGAAGGGGGAAGAATTCCTGGTGTAGCGATGAAATGCGTAGAGATCTGGAGGAATACCGGTGGCGAAGG

CGGCCCCCTGGACAAAGACTGACGCTCAGGTGCGAAAGCGTGGGGAGCAAACAGGATTAGATACCCTGGT

AGTCCACGCCGTAAACGATGTCGATTTGGAGGCTGTGTCCTTGAGACGTGGCTTCCGGAGCTAACGCGTT

AAATCGACCGCCTGGGGAGTACGGCCGCAAGGTTAAAACTCAAATGAATTGACGGGGGCCCGCACAAGCG

GTGGAGCATGTGGTTTAATTCGATGCAACGCGAAGAACCTTACCTGGCCTTGACATGTCTGGAATCCTGC

AGAGATGCGGGAGTGCCTTCGGGAATCAGAACACAGGTGCTGCATGGCTGTCGTCAGCTCGTGTCGTGAG

ATGTTGGGTTAAGTCCCGCAACGAGCGCAACCCCTGTCCTTTGTTGCCAGCACGTAATGGTGGGAACTCA

AGGGAGACTGCCGGTGATAAACCGGAGGAAGGTGGGGATGACGTCAAGTCATCATGGCCCTTACGGCCAG

GGCTACACACGTGCTACAATGGCGCGTACAGAGGGCTGCAAGCTAGCGATAGTGAGCGAATCCCAAAAAG

CGCGTCGTAGTCCGGATCGGAGTCTGCAACTCGACTCCGTGAAGTCGGAATCGCTAGTAATCGCAAATCA

GAATGTTGCGGTGAATACGTTCCCGGGCCTTGTACACACCGCCCGTCACACCATGGGAGTGGGTTGCACC

AGAAGTAGATAGCTTAACCTTCGGGAGGGCGTTACCACGGGTGATTCG

**MT396437**

AGATTGAACGCTGGCGGCAGGCCTAACACATGCAAGTCGAGCGGCAGCGGGAAAGTAGCTTGCTACTTTT

GCCGGCGAGCGGCGGACGGGTGAGTAATGCCTGGGAAATTGCCCAGTCGAGGGGGATAACAGTTGGAAAC

GACTGCTAATACCGCATACGCCCTACGGGGGAAAGCAGGGGACCTTCGGGCCTTGCGCGATTGGATATGC

CCAGGTGGGATTAGCTAGTTGGTGAGGTAATGGCTCACCAAGGCGACGATCCCTAGCTGGTCTGAGAGGA

TGATCAGCCACACTGGAACTGAGACACGGTCCAGACTCCTACGGGAGGCAGCAGTGGGGAATATTGCACA

ATGGGGGAAACCCTGATGCAGCCATGCCGCGTGTGTGAAGAAGGCCTTCGGGTTGTAAAGCACTTTCAGC

GAGGAGGAAAGGTTGATGCCTAATACGTATCGACTGGGACGTTACTCGCAGAAGAAGCACCGGCTAACTC

CGTGCCAGCAGCCGCGGTAATACGGAGGGTGCAAGCGTTAATCGGAATTACTGGGCGTAAAGCGCACGCA

GGCGGTGCGATACAGTTATATGTGAAAGCCCCGGGCTCAACCTGGGAATTGCATTTAAAACTGTTCAGCT

AGAGTTCTTGTAGAGGGGGGTAGAATTCCAGGTGTAGCGGTGAAATGCGTAGAGATCTGGAGGAATACCG

GTGGCGAAGGCGGCCCCCTGGACAAAGACTGTCGCTCAGGTGCGAAAGCGTGGGGAGCAAACAGGATTAG

ATACCCTGGTAGTCCACGCCGTAAACGATGTCGATTTGGAGGCTGTGTCCTTGAGACGTGGCTTCCGGAG

CTAACGCGTTAAATCGACCGCCTGGGGAGTACGGCCGCAAGGTTAAAACTCAAATGAATTGACGGGGGCC

CGCACAAGCGGTGGAGCATGTGGTTTAATTCGATGCAACGCGAAGAACCTTACCTGGCCTTGACATGTCT

GGAATCCTGCAGAGATGCGGGAGTGCCTTCGGGAATCAGAACACAGGTGCTGCATGGCTGTCGTCAGCTC

GTGTCGTGAGATGTTGGGTTAAGTCCCGCAACGAGCGCAACCCCTGTCCTTTGTTGCCAGCACGTAATGG

TGGGAACTCAAGGGAGACTGCCGGTGATAAACCGGAGGAAGGTGGGGATGACGTCAAGTCATCATGGCCC

TTACGGCCAGGGCTACACACGTGCTACAATGGCGCGTACAGAGGGCTGCAAGCTAGCGATAGTGAGCGAA

TCCCAAAAAGCGCGTCGTAGTCCGGATCGGAGTCTGCAACTCGACTCCGTGAAGTCGGAATCGCTAGTAA

TCGCAAATCAGAATGTTGCGGTGAATACGTTCCCGGGGCCTTGTACACACCGCCCGTCACACCATGGGGA

GTGGGGTTGCACCAGAAAGTAGATAGCTTAACCTTCGGGGAGGGCGTTTACCACGGTGTGATTCATGACT

GGGGGTGAAGTCGTAACAAGGTAACCCTAGGGG

**MT396438**

ATGGCGGGCGGCAGGCCTAACACATGCAAGTCGAGCGGCAGCGGGAAAGTAGCTTGCTACTTTTGCCGGC

GAGCGGCGGACGGGTGAGTAATGCCTGGGGATCTGCCCAGTCGAGGGGGATAACTACTGGAAACGGTAGC

TAATACCGCATACGCCCTACGGGGGAAAGCAGGGGACCTTCGGGCCTTGCGCGATTGGATGAACCCAGGT

GGGATTAGCTAGTTGGTGAGGTAATGGCTCACCAAGGCGACGATCCCTAGCTGGTCTGAGAGGATGATCA

GCCACACTGGAACTGAGACACGGTCCAGACTCCTACGGGAGGCAGCAGTGGGGAATATTGCACAATGGGG

GAAACCCTGATGCAGCCATGCCGCGTGTGTGAAGAAGGCCTTCGGGTTGTAAAGCACTTTCAGCGAGGAG

GAAAGGTTGGTAGCTAATAACTGCCAGCTGTGACGTTACTCGCAGAAGAAGCACCGGCTAACTCCGTGCC

AGCAGCCGCGGTAATACGGAGGGTGCAAGCGTTAATCGGAATTACTGGGCGTAAAGCGCACGCAGGCGGT

TGGATAAGTTAGATGTGAAAGCCCCGGGCTCAACCTGGGAATTGCATTTAAAACTGTCCAGCTAGAGTCT

TGTAGAGGGGGGTAGAATTCCAGGTGTAGCGGTGAAATGCGTAGAGATCTGGAGGAATACCGGTGGCGAA

GGCGGCCCCCTGGACAAAGACTGACGCTCAGGTGCGAAAGCGTGGGGAGCAAACAGGATTAGATACCCTG

GTAGTCCACGCCGTAAACGATGTCGATTTGGAGGCTGTGTCCTTGAGACGTGGCTTCCGGAGCTAACGCG

TTAAATCGACCGCCTGGGGAGTACGGCCGCAAGGTTAAAACTCAAATGAATTGACGGGGGCCCGCACAAG

CGGTGGAGCATGTGGTTTAATTCGATGCAACGCGAAGAACCTTACCTGGCCTTGACATGTCTGGAATCCT

GTAGAGATACGGGAGTGCCTTCGGGAATCAGAACACAGGTGCTGCATGGCTGTCGTCAGCTCGTGTCGTG

AAATGTTGGGTTAAGTCCCGCAACGAGCGCAACCCCTGTCCTTTGTTGCCAGCACGTAATGGTGGAAACT

CAAGGGAAACTCCCCGTGATAAACCGGAGGAAGGTGGGGATGACGTCAAGTCATCATGGCCCTTACGGCC

AGGGCTACACACGTGCTACAATGGCGCGTACCAGAGGGCTGCAAGCTAGCGATAGTGAGCGAATCCCAAA

AAGCGCGTCGTAGTCCGGATCGGAGTCTGCAACTCGACTCCGTGAAGTCGGAATCGCTAGTAATCGCAAA

TCAGAATGTTGCGGTGAATACGTTCCCGGGCCTTGTACACACCGCCCGTCACACCATGGGAGTGGGTTGC

ACCAGAAGTAGATAG

**MT393942**

TGCAGTCGAGCGGCAGCGGGAAAGTAGCTTGCTACTTTTGCCGGCGAGCGGCGGACGGGTGAGTAATGCC

TGGGAAATTGCCCAGTCGAGGGGGATAACAGTTGGAAACGACTGCTAATACCGCATACGCCCTACGGGGG

AAAGCAGGGGACCTTCGGGCCTTGCGCGATTGGATATGCCCAGGTGGGATTAGCTAGTTGGTGAGGTAAT

GGCTCACCAAGGCGACGATCCCTAGCTGGTCTGAGAGGATGATCAGCCACACTGGAACTGAGACACGGTC

CAGACTCCTACGGGAGGCAGCAGTGGGGAATATTGCACAATGGGGGAAACCCTGATGCAGCCATGCCGCG

TGTGTGAAGAAGGCCTTCGGGTTGTAAAGCACTTTCAGCGAGGAGGAAAGGTCAGTAGCTAATATCTGCT

GGCTGTGACGTTACTCGCAGAAGAAGCACCGGCTAACTCCGTGCCAGCAGCCGCGGTAATACGGAGGGTG

CAAGCGTTAATCGGAATTACTGGGCGTAAAGCGCACGCAGGCGGTTGGATAAGTTAGATGTGAAAGCCCC

GGGCTCAACCTGGGAATTGCATTTAAAACTGTCCAGCTAGAGTCTTGTAGAGGGGGGTAGAATTCCAGGT

GTAGCGGTGAAATGCGTAGAGATCTGGAGGAATACCGGTGGCGAAAGGCGGCCCCCTGGACAAAGACTGA

CGCTCAGTGCGAAAGCGTGGGGAGCAAACAGGATTAGATACCCTGGTAGTCCACGCCGTAAACGATGTCG

ATTTGGAGGCTGTGTCCTTGAGACGTGGCTTCCGGAGCTAACGCGTTAAATCGACCG

**MT393943**

TCAGATTGAACGCTGGCGGCAGGCCTAACACATGCAAGTCGAGCGGCAGCGGGAAAGTAGCTTGCTACTT

TTGCCGGCGAGCGGCGGACGGGTGAGTAATGCCTGGGAAATTGCCCAGTCGAGGGGGATAACAGTTGGAA

ACGACTGCTAATACCGCATACGCCCTACGGGGGAAAGCAGGGGACCTTCGGGCCTTGCGCGATTGGATAT

GCCCAGGTGGGATTAGCTAGTTGGTGAGGTAATGGCTCACCAAGGCGACGATCCCTAGCTGGTCTGAGAG

GATGATCAGCCACACTGGAACTGAGACACGGTCCAGACTCCTACGGGAGGCAGCAGTGGGGAATATTGCA

CAATGGGGGAAACCCTGATGCAGCCATGCCGCGTGTGTGAAGAAGGCCTTCGGGTTGTAAAGCACTTTCA

GCGAGGAGGAAAGGTCAGTAGCTAATATCTGCTGGCTGTGACGTTACTCGCAGAAGAAGCACCGGCTAAC

TCCGTGCCAGCAGCCGCGGTAATACGGAGGGTGCAAGCGTTAATCGGAATTACTGGGCGTAAAGCGCACG

CAGGCGGTTGGATAAGTTAGATGTGAAAGCCCCGGGCTCAACCTGGGAATTGCATTTAAAACTGTCCAGC

TAGAGTCTTGTAGAGGGGGGTAGAATTCCAGGTGTAGCGGTGAAATGCGTAGAGATCTGGAGGAATACCG

GTGGCGAAGGCGGCCCCCTGGACAAAGACTGACGCTCAGGTGCGAAAGCGTGGGGAGCAAACAGGATTAG

ATACCCTGGTAGTCCACGCCGTAAACGATGTCGATTTGGAGGCTGTGTCCTTGAGACGTGGCTTCCGGAG

CTAACGCGTTAAATCGACCGCCTGGGGAGTACGGCCGCAAGGTTAAAACTCAAATGAATTGACGGGGGCC

CGCACAAGCGGTGGAGCATGTGGTTTAATTCGATGCAACGCGAAGAACCTTACCTGGCCTTGACATGTCT

GGAATCCTGCAGAGATGCGGGAGTGCCTTCGGGAATCAGAACACAGGTGCTGCATGGCTGTCGTCAGCTC

GTGTCGTGAGATGTTGGGTTAAGTCCCGCAACGAGCGCAACCCCTGTCCTTTGTTGCCAGCACGTAATGG

TGGGAACTCAAGGGAGACTGCCGGTGATAAACCGGAGGAAGGTGGGGATGACGTCAAGTCATCATGGCCC

TTACGGCCAAGGCTACACACGTGCTACATGGCCGTACGAAGGCTGCAGCTTAGCGATAGTGAGCGAATCC

AAAAAGCGCGTCGTAGTCCGGATTGGAGTCTGCAACTCGACTCCGTGAAGTCGGAATCGCTAGTAATCGC

AAATCAGAATGTTGCGGTGAATACGTTCCCGGGCCTTGTACACACCGCCCGTCACA

**MT393930**

GCTACACATGCAAGTCGAGCGGCAGCGGGAAAGTAGCTTGCTACTTTTGCCGGCGAGCGGCGGACGGGTG

AGTAATGCCTGGGAAATTGCCCAGTCGAGGGGGATAACAGTTGGAAACGACTGCTAATACCGCATACGCC

CTACGGGGGAAAGCAGGGGACCTTCGGGCCTTGCGCGATTGGATATGCCCAGGTGGGATTAGCTAGTTGG

TGAGGTAATGGCTCACCAAGGCGACGATCCCTAGCTGGTCTGAGAGGATGATCAGCCACACTGGAACTGA

GACACGGTCCAGACTCCTACGGGAGGCAGCAGTGGGGAATATTGCACAATGGGGGAAACCCTGATGCAGC

CATGCCGCGTGTGTGAAGAAGGCCTTCGGGTTGTAAAGCACTTTCAGCGAGGAGGAAAGGTCGGTGGCTA

ATATCTGCTGGCTGTGACGTTACTCGCAGAAGAAGCACCGGCTAACTCCGTGCCAGCAGCCGCGGTAATA

CGGAGGGTGCAAGCGTTAATCGGAATTACTGGGCGTAAAGCGCACGCAGGCGGTTGGATAAGTTAGATGT

GAAAGCCCCGGGCTCAACCTGGGAATTGCATTTAAAACTGTCCAGCTAGAGTCTTGTAGAGGGGGGTAGA

ATTCCAGGTGTAGCGGTGAAATGCGTAGAGATCTGGAGGAATACCGGTGGCGAAAGCGGCCCCCTGGACA

AAGACTGACGCTCAGGTGCGAAAGCGTGGGGAGCAAACAGGATTAGATACCCTGGTAGTCCACGCCGTAA

ACGATGTCGATTTGGAGGCTGTGTCCTTGAAACGGGGCTTCCGGAGCTAACCGCGTTAAATCGACCGCCT

GGGG

**MT393932**

TGCAAGTCGAGCGGCAGCGGGAAAGTAGCTTGCTACTTTTGCCGGCGAGCGGCGGACGGGTGAGTAATGC

CTGGGAAATTGCCCAGTCGAGGGGGATAACAGTTGGAAACGACTGCTAATACCGCATACGCCCTACGGGG

GAAAGCAGGGGACCTTCGGGCCTTGCGCGATTGGATATGCCCAGGTGGGATTAGCTAGTTGGTGAGGTAA

TGGCTCACCAAGGCGACGATCCCTAGCTGGTCTGAGAGGATGATCAGCCACACTGGAACTGAGACACGGT

CCAGACTCCTACGGGAGGCAGCAGTGGGGAATATTGCACAATGGGGGAAACCCTGATGCAGCCATGCCGC

GTGTGTGAAGAAGGCCTTCGGGTTGTAAAGCACTTTCAGCGAGGAGGAAAGGTCGTTGGCTAATATCTGA

TGGCTGTGACGTTACTCGCAGAAGAAGCACCGGCTAACTCCGTGCCAGCAGCCGCGGTAATACGGAGGGT

GCAAGCGTTAATCGGAATTACTGGGCGTAAAGCGCACGCAGGCGGTTGGATAAGTTAGATGTGAAAGCCC

CGGGCTCAACCTGGGAATTGCATTTAAAACTGTCCAGCTAGAGTCTTGTAGAGGGGGGTAGAATTCCAGG

TGTAGCGGTGAAATGCGTAGAGATCTGGAGGAATACCGGTGGCGAAGGCGGCCCCCTGGACAAAGACTGA

CGCTCAGGTGCGAAAGCGTGGGGAGCAAACAGGATTAGATACCCTGGTAGTCCACGCCGTAAACGATGTC

GATTTGGAGGCTGTGTCCTTGAGACGTGGCTTCCGGAGCTAACGCGTTAAATCGACCGCCTGGGGAGTAC

GGCCGCAAGGTTAAAACTCAAATGAATTGACGGGGGCCCGCACAAGCGGTGGAGCATGTGGTTTAATTCG

ATGCAACGCGAAGAACCTTACCTGGCCTTGACATGTCTGGAATCCTGTAGAGATACGGGAGTGCCTTCGG

GAATCAGAACACAGGTGCTGCATGGCTGTCGTCAGCTCGTGTCGTGAGATGTTGGGTTAAGTCCCGCAAC

GAGCGCAACCCCTGTCCTTTGTTGCCGCACGTAATGGTGGAACTCAAGGAGACTGCCGTGATAAACCGAA

GAAGGGGGGATGACGTCAGTCTTCTGGCCTTACGCCAGGGCTACACACGTGCTACAATGGCGCGTACAGA

GGGCTGCAAGCTAGCGATAGTGAGCGAATCCCAAAAAGCGCGTCGTAGTCCGGATTGGAGTCTGCAACTC

GACTCCATGAAGTCGGAATCGCTAGTAATCGCAAATCAGAATGTTGCGGTGAATACGTTCCCGGGCCTTG

TACACACCGCCCGTCACACCATGGGAGTGGGTTGCACCAGAAGTAGATAGCTTAACCTTCGGGAGGGCGT

T

**MT397061**

GATTGCGTCCGCAGCCTTCACATGCAGTTCGAGCGGCAGCGGGAAAGTTAGCTTGCTACTTTTTGCCGGC

GAGCGGCGGACGGGTGAGTAATGCCTGGGGATCTGCCCAGTCGAGGGGGATAACTACTGGAAACGGTAGC

TAATACCGCATACGCCCTACGGGGGAAAGCAGGGGACCTTCGGGCCTTGCGCGATTGGATGAACCCAGGT

GGGATTAGCTAGTTGGTGAGGTAATGGCTCACCAAGGCGACGATCCCTAGCTGGTCTGAGAGGATGATCA

GCCACACTGGAACTGAGACACGGTCCAGACTCCTACGGGAGGCAGCAGTGGGGAATATTGCACAATGGGG

GAAACCCTGATGCAGCCATGCCGCGTGTGTGAAGAAGGCCTTCGGGTTGTAAAGCACTTTCAGCGAGGAG

GAAAGGTTGGTAGCTAATAACTGCCAGCTGTGACGTTACTCGCAGAAGAAGCACCGGCTAACTCCGTGCC

AGCAGCCGCGGTAATACGGAGGGTGCAAGCGTTAATCGGAATTACTGGGCGTAAAGCGCACGCAGGCGGT

TGGATAAGTTAGATGTGAAAGCCCCGGGCTCAACCTGGGAATTGCATTTAAAACTGTCCAGCTAGAGTCT

TGTAGAGGGGGGTAGAATTCCAGGTGTAGCGGTGAAATGCGTAGAGATCTGGAGGAATACCGGTGGCGAA

GGCGGCCCCCTGGACAAAGACTGACGCTCAGGTGCGAGAGCGTGGGGAGCAAACAGGATTAGATACCCTG

GTAGTCCACGCCGTAAACGATGTCGATTTGGAGGCTGTGTCCTTGAGACGTGGCTTCCGGAGCTAACGCG

TTAAATCGACCGCCTGGGGAGTACGGCCGCAAGGTTAAAACTCAAATGAATTGACGGGGGCCCGCACAAG

CGGTGGAGCATGTGGTTTAATTCAATGCAACGCGAAGAACCTTACCTGGCCTTGACATGTCTGGAATCCT

GCAAAGATGCGGGAATGCCTT

**MT397058**

AGCGCATTGCCGCAGCCTCACATGCAGTCCGAGCGGCAGCGGGAAAGTTAGCTTGCTTACTTTATTTGCC

GGCGAGCGGCGGACGGGTGAGTAATGCCTGGGGATCTGCCCAGTCGAGGGGGATAACTACTGGAAACGGT

AGCTAATACCGCATACGCCCTACGGGGGAAAGCAGGGGACCTTCGGGCCTTGCGCGATTGGATGAACCCA

GGTGGGATTAGCTAGTTGGTGAGGTAATGGCTCACCAAGGCGACGATCCCTAGCTGGTCTGAGAGGATGA

TCAGCCACACTGGAACTGAGACACGGTCCAGACTCCTACGGGAGGCAGCAGTGGGGAATATTGCACAATG

GGGGAAACCCTGATGCAGCCATGCCGCGTGTGTGAAGAAGGCCTTCGGGTTGTAAAGCACTTTCAGCGAG

GAGGAAAGGTTGGTAGCTAATAACTGCCAGCTGTGACGTTACTCGCAGAAGAAGCACCGGCTAACTCCGT

GCCAGCAGCCGCGGTAATACGGAGGGTGCAAGCGTTAATCGGAATTACTGGGCGTAAAGCGCACGCAGGC

GGTTGGATAAGTTAGATGTGAAAGCCCCGGGCTCAACCTGGGAATTGCATTTAAAACTGTCCAGCTAGAG

TCTTGTAGAGGGGGGTAGAATTCCAGGTGTAGCGGTGAAATGCGTAGAGATCTGGAGGAATACCGGTGGC

GAAGGCGGCCCCCTGGACAAAGACTGACGCTCAGGTGCGAAAGCGTGGGGAGCAAACAGGATTAGATACC

CTGGTAGTCCACGCCGTAAACGATGTCGATTTGGAGGCTGTGTCCTTGAGACGTGGCTTCCGGAGCTAAC

GCGTTAAATCGACCGCCTGGGGAGTACGGCCGCAAGGTTAAAACTCAAATGAATTGACGGGGGCCCGCAC

AAGCGGTGGAGCATGTGGTTTAATTCGATGCAACGCGAAGAACCTTACCTGGCCTTGACATGTCTGGAAT

CCTGTAAAGATACGGGAGTGC

**MT397063**

GGTTGACGGCCGCCGCTAAACATGCAGTTCGAGCCGCAGCCGGGAAAGTAGCTTGCTACTTATTGCCGGC

GAGCGGCGGACGGGTGAGTAATGCCTGGGGATCTGCCCAGTCGAGGGGGATAACTACTGGAAACGGTAGC

TAATACCGCATACGCCCTACGGGGGAAAGCAGGGGACCTTCGGGCCTTGCGCGATTGGATGAACCCAGGT

GGGATTAGCTAGTTGGTGAGGTAATGGCTCACCAAGGCGACGATCCCTAGCTGGTCTGAGAGGATGATCA

GCCACACTGGAACTGAGACACGGTCCAGACTCCTACGGGAGGCAGCAGTGGGGAATATTGCACAATGGGG

GAAACCCTGATGCAGCCATGCCGCGTGTGTGAAGAAGGCCTTCGGGTTGTAAAGCACTTTCAGCGAGGAG

GAAAGGTTGGTAGCTAATAACTGCCAGCTGTGACGTTACTCGCAGAAGAAGCACCGGCTAACTCCGTGCC

AGCAGCCGCGGTAATACGGAGGGTGCAAGCGTTAATCGGAATTACTGGGCGTAAAGCGCACGCAGGCGGT

TGGATAAGTTAGATGTGAAAGCCCCGGGCTCAACCTGGGAATTGCATTTAAAACTGTCCAGCTAGAGTCT

TGTAGAGGGGGGTAGAATTCCAGGTGTAGCGGTGAAATGCGTAGAGATCTGGAGGAATACCGGTGGCGAA

GGCGGCCCCCTGGACAAAGACTGACGCTCAGGTGCGAAAGCGTGGGGAGCAAACAGGATTAGATACCCTG

GTAGTCCACGCCGTAAACGATGTCGATTTGGAGGCTGTGTCCTTGAGACGTGGCTTCCGGAGCTAACGCG

TTAAATCGACCGCCTGGGGAGTACGGCCGCAAGGTTAAGACTCAAATGAATTGACGGGGGCCCGCACAAG

CGGTGGAGCATGTGGTTTAATTCAATGCAACGCGGAGAACCTTACCTGGCCTTGACATGTCTGGAATCCT

GTAAAGATACGGGAGTGCCTTCGGGAATCAGAACACAGGTGCTGCATGGCTGTCGTCAGCTCCTGTCCTG

AGATGTTGGGTTAATTCCCCCAACGAAGGCAACCCCTGTCCTTTGTTGCCAGCACGTAATGGTGGGAACT

CAAGGGAGACTGCCGGTGATAAACCGGAGGAAGGTGGGGATGACGTCAAGTCATCATGGCCCTTACGGCC

AGGGCTACACACGTGCTACAATGGCGCGTACAGAGGGCTGCAAGCTAGCGATAGTGAGCGAATCCCAAAA

AGCGCGTCGTAGTCCGGATCGGAGTCTGCAACTCGACTCCGTGAAGTCGGAATCGCTAGTAATCGCAAAT

CAGAATGTTGCGGTGAATACGTTCCCGGGCCTTGTACACACCGCCCGTCACACCATGGGAGTGGGTTGCA

CCAGAAGTAGATAGCTTAACCTTCGGGAGGGCGTTACCATCGGTG

**MT704303**

CTGAGCGAGAAGGTGACAACCAAGAACAAGTTTCAGTGGCCGTTGGTGGGGGAAACCGAACTGGCTATCG

AGATCGCTGCGAGCCAGAGCTGGGCTTCCCAGAAAGGGGGCTCGACGACCGAGACCGTCTCGGTTGAAGC

GCGCCCCACGGTGCCGCCTCACTCCAGCCTGCCGGTGCGGGTTGCCCTCTACAAGTCCAACATCTCCTAC

CCCTACGAGTTCAAGGCCGAGGTCAATTATGACCTGACCATGAAGGGCTTCCTGCGTTGGGGCGGCAATG

CCTGGTATACCCATCCGGAGAATCGTCCCACCTGGGAGCACACCTTTGCCGTCGGCCCGTTCCGCGACAA

GGCGAGCAGCATCCGCTACCAGTGGGACAAGCGTTATATCCCGGGTGAAGTGAAGTGGTGGGACTGGAAC

TGG

**MT704304**

TATGGCCTTAGCGAGAAGGTCACCACCAAGAACAAGTTTCAGTGGCCGTTGGTGGGGGAAACCGAACTGG

CCATCGAGATCGCTGCGAGCCAGAGCTGGGCTTCCCAGAAAGGGGGCTCAACGACCGAGACCGTCTCGGT

TGAAGCGCGCCCCACGGTGCCGCCTCACTCCAGCCTGCCGGTGCGGGTTGCCCTCTACAAGTCCAACATC

TCCTATCCCTACGAGTTCAAGGCCGAGGTCAATTATCACCTCACCATTAAGGGCTTCCTGCGTTGGGGCG

GCAATGCCTGGTATACCCATCCGGAGAATCGCCCCACCTGGGAGCACACCTTTGCCGTCGGCCCGTTCCG

CGACAAGGCCAGCAGCATTCGCTACCAGTGGGACAAGCGTTATATCCCGGGTGAAGTGAAGTGGTGGGAC

TGGAACTGG

**MT704305**

AGCCTGAGCGAGAAGGTGACCACCAAGAACAAGTTTCAGTGGCCGTTGGTGGGGGAAACCGAACTGGCCA

TCGAGATCGCTGCGAGCCAGAGCTGGGCTTCCCAGAAAGGGGGCTCGACGACCGAGACCGTCTCGGTTGA

AGCGCGCCCCACGGTGCCGCCTCACTCCAGCCTGCCGGTGCGGGTTGCCCTCTACAAGTCCAACATCTCC

TACCCCTACGAGTTCAAGGCCGAGGTCAATTATGACCTGACCATGAAGGGCTTCCTGCGTTGGGGCGGCA

ATGCCTGGTATACCCATCCGGAGAATCGCCCCACCTGGGAGCACACCTTTGCCGTCGGCCCGTTCCGCGA

CAAGGCGAGCAGCATCCGCTACCAGTGGGACAAGCGTTATATCCCGGGTGAAGTGAAGTGGTGGGACTGG

AACTGGACC

**MT704306**

CTTGGCGAGAAGGCGACCACCAAGAACAAGTTTCAGTGGCCGTTGGTGGGGGAAACCGAACTGGCCATCG

AGATCGCTGCGAGCCAGAGCTGGGCTTCCCAGAAAGGGGGCTCAACGACCGAGACCGTCTCGGTGGAAGC

GCGCCCCACGGTGCCGCCTCACTCCAGCCTGCCGGTGCGGGTTGCCCTCTACAAGTCCAACATCTCCTAC

CCCTACGAGTTGAAGGCCGAGGTCAATTATGTCCTTACCACGAAGGGCTTCCTGCGTTGGGGCGGCAATG

CCTGGTATACCCATCCGGAGAATCGCCCCACCTGGGAGCACACCTTTGCCGTCGGCCCGTTCCGCGACAA

GGCGAGCAGCATCCGCTATCAGTGGGACAAGCGTTATATCCCGGGTGAAGTTAGGTGGTGGGACTGGAAC

TGG

**MT704307**

TATGGCCTTAGCGAGAAGGTCACCACCAAGAACAAGTTTCAGTGGCCGTTGGTGGGGGAAACCGAACTGG

CCATCGAGATCGCTGCGAGCCAGAGCTGGGCTTCCCAGAAAGGGGGCTCAACGACCGAGACCGTCTCGGT

TGAAGCGCGCCCCACGGTGCCGCCTCACTCCAGCCTGCCGGTGCGGGTTGCCCTCTACAAGTCCAACATC

TCCTACCCCTACGAGTTCAAGGCCGAGGTCAATTATCACCTCACCATTAAGGGCTTCCTGCGTTGGGGCG

GCAATGCCTGGTATACCCATCCGGAGAATCGTCCCACCTGGGAGCACACCTTTGCCGTCGGCCCGTTCCG

CGACAAGGCGAGCAGCATCCGCTACCAGTGGGACAAGCGTTATATCCCGGGTGAAGTCAAGTGGTGGGAC

TGGAACTGG

**MT704308**

CTGAGCGAGAAGGTGACAACCAAGAACAAGTTTCAGTGGCCGTTGGTGGGGGAAACCGAACTGGCCATCG

AGATCGCTGCGAGCCAGAGCTGGGCCTCCCAGAAAGGGGGCTCGACGACCGAGACCGTCTCGGTTGAAGC

GCGCCCCACGGTGCCGCCTCACTCCAGCCTGCCGGTGCGGGTTGCCCTCTACAAGTCCAACATCTCCTAC

CCCTACGAGTTCAAGGCCGAGGTCAATTATGACCTGACCATGAAGGGCTTCCTGCGTTGGGGCGGCAATG

CCTGGTATACCCATCCGGAGAATCGTCCCACCTGGGAGCACACCTTTGCCGTCGGCCCGTTCCGTGACAA

GGCGAGCAGCATCCGCTACCAGTGGGACAAGCGTTATATCCCGGGTGAAGTGAAGTGGTGGGACTGGAAC

TGG

**MT704309**

CTGAGCGAGAAGGTGACAACCAAGAACAAGTTTCAGTGGCCGTTGGTGGGGGAAACCGAACTGGCTATCG

AGATCGCTGCGAGCCAGAGCTGGGCTTCCCAGAAAGGGGGCTCGACGACCGAGACCGTCTCGGTTGAAGC

GCGCCCCACGGTGCCGCCCCACTCCAGCCTGCCGGTGCGGGTTGCCCTCTACAAGTCCAACATCTCCTAC

CCCTACGAGTTCAAGGCCGAGGTCAATTATGACCTGACCATGAAGGGCTTCCTGCGTTGGGGCGGCAATG

CCTGGTATACCCATCCGGAGAATCGTCCCACCTGGGAGCACACCTTTGCCGTCGGCCCGTTCCGCGACAA

GGCGAGCAGCATCCGCTACCAGTGGGACAAGCGTTATATCCCGGGTGAAGTGAAGTGGTGGGACTGGAAC

TGG

**MT707932**

CTGAGCGAGAAGGTGACAACCAAGAACAAGTTTCAGTGGCCGTTGGTGGGGGAAACCGAACTGGCTATCG

AGATCGCTGCGAGCCAGAGCTGGGCCTCCCAGAAAGGGGGCTCGACGACCGAGACCGTCTCGGTTGAAGC

GCGCCCCACGGTGCCGCCTCACTCCAGCCTGCCGGTGCGGGTTGCCCTCTACAAGTCCAACATCTCCTAC

CCCTACGAGTTCAAGGCCGAGGTCAATTATGACCTGACCATGAAGGGCTTCCTGCGTTGGGGCGGCAATG

CCTGGTATACCCATCCTGAGAATCGTCCCACCTGGGAGCACACCTTTGCCGTCGGCCCGTTCCGCGACAA

GGCGAGCAGCATCCGCTACCAGTGGGACAAGCGTTATATCCCGGGTGAAGTGAAGTGGTGGGACTGGAAC

TGG

**MH607886**

CTGAGCGAGAAGGTGACAACCAAGAACAAGTTTCAGTGGCCGTTGGTGGGGGAAACCGAACTGGCTATCG

AGATCGCTGCGAGCCAGAGCTGGGCTTCCCAGAAAGGGGGCTCGACGACCGAGACCGTCTCGGTTGAAGC

GCGCCCCACGGTGCCGCCCCACTCCAGCCTGCCGGTGCGGGTTGCCCTCTACAAGTCCAACATCTCCTAC

CCCTACGAGTTCAAGGCCGAGGTCAATTATGACCTGACCATGAAGGGCTTCCTGCGTTGGGGCGGCAATG

CCTGGTATACCCATCCGGAGAATCGTCCCACCTGGGAGCACACCTTTGCCGTCGGCCCGTTCCGCGACAA

GGCGAGCAGCATCCGCTACCAGTGGGACAAGCGCTATATCCCGGGTGAAGTGAAGTGGTGGGACTGGAAC

TGG

**MT707935**

TCCTACAGCCTTGGCGAGAAGGCGACCACCAAGAACAAGTTTCAGTGGCCGTTGGTGGGGGAAACCGAAC

TGGCCATCGAGATCGCTGCGAGCCAGAGCTGGGCTTCCCAGAAAGGGGGCTCAACGACCGAGACCGTCTC

GGTGGAAGCGCGCCCCACGGTGCCGCCTCACTCCAGCCTGCCGGTGCGGGTTGCCCTCTACAAGTCCAAC

ATCTCCTACCCCTACGAGTTGAAGGCCGAGGTCAATTATGTCCTTACCACGAAGGGCTTCCTGCGTTGGG

GCGGCAATGCCTGGTATACCCATCCGGAGAATCGTCCCACCTGGGAGCACACCTTTGCCGTCGGCCCGTT

CCGCGACAAGGCGAGCAGCATCCGCTACCAGTGGGACAAGCGTTATATCCCGGGTGAAGTGAGGTGGTGG

GACTGGAACTG

**MH591426**

AGCCTGAGCGAGAAGGTGACCACCAAGAACAAGTTTCAGTGGCCGTTGGTGGGGGAAACCGAACTGGCCA

TCGAGATCGCTGCGAGCCAGAGTTGGGCCTCCCAGAAAGGGGGCTCGACGACCGAGACCGTCTCGGTTGA

AGCGCGCCCCACGGTGCCGCCTCACTCCAGCCTTCCGGTGCGGGTTGCCCTCTACAAGTCCAACATCTCC

TACCCCTACGAGTTCAAGGCCGAGGTCAATTATGACCTGACCATGAAGGGCTTCCTGCGTTGGGGCGGCA

ATGCCTGGTATACCCATCCTGAGAATCGCCCCACCTGGGAGCACACCTTTGCCGTCGGCCCGTTCCGCGA

CAAGGCCAGCAGCATCCGCTACCAGTGGGACAAGCGTTATATCCCGGGTGAAGTGAAGTGGTGGGACTGG

AACTGGACC

**MT813045**

TCCTACAGCCTTGGCGAGAAGGCGACCACCAAGAACAAGTTTCAGTGGCCGTTGGTGGGGGAAACCGAAC

TGGCCATCGAGATCGCTGCGAGCCAGAGCTGGGCTTCCCAGAAAGGGGGCTCAACGACCGAGACCGTCTC

GGTGGAAGCGCGCCCCACGGTGCCGCCTCACTCCAGCCTGCCGGTGCGGGTTGCCCTCTACAAGTCCAAC

ATCTCCTACCCCTACGAGTTGAAGGCCGAGGTCAATTATGTCCTTACCACGAAGGGCTTCCTGCGTTGGG

GCGGCAATGCCTGGTATACCCATCCGGAGAATCGCCCCACCTGGGAGCACACCTTTGCCGTCGGCCCGTT

CCGCGACAAGGCGAGCAGCATCCGCTATCAGTGGGACAAGCGTTATATCCCGGGTGAAGTTAGGTGGTGG

GACTGGAACTGG

**MT707933**

AGCGAGAAGGTGACTACCAAGAACAAGTTTCAGTGGCCGCTGGTGGGTGAAACCGAACTCGCCATCGAGA

TCGCTGCGAGCCAGAGCTGGGCCTCCCAGAAAGGGGGCTCGACGACCGAGACCGTCTCGGTTGAAGCGCG

CCCCACGGTGCCGCCCCACTCCAGCCTGCCGGTGCGGGTTGCCCTCTACAAGTCCAACATCTCCTACCCC

TACGAGTTCAAGGCCGAGATCAATTATGACCTGACCATGAAGGGCTTCCTGCGTTGGGGCGGCAATGCCT

GGTATACCCATCCTGAGAATCGTCCCACCTGGGAGCACACCTTTGCCGTAGGCCCGTTCCGCGACAAGGC

GAGCAGCATCCGTTATCAGTGGGACAAGCGTTATATCCCGGGTGAAGTGAAGTGGTGG

**MT707934**

TATGGCCTGAGCGAGAAGGTGACAACCAAGAACAAGTTTCAGTGGCCGTTGGTGGGGGAAACCGAACTGG

CCATCGAGATCGCTGCGAGCCAGAGCTGGGCTTCCCAGAAAGGGGGCTCGACGACCGAGACCGTCTCGGT

TGAAGCGCGCCCCACGGTGCCGCCTCACTCCAGCCTGCCGGTGCGGGTTGCCCTCTACAAGTCCAACATC

TCCTACCCCTACGAGTTCAAGGCCGAGGTCAATTATCACCTGACCATTAAGGGCTTCCTGCGTTGGGGCG

GCAATGCCTGGTATACCCATCCGGAGAATCGTCCCACCTGGGAGCACACCTTTGCCGTCGGCCCGTTCCG

CGACAAGGCGAGCAGCATCCGCTACCAGTGGGACAAGCGTTATATCCCGGGTGAAGTGAAGTGGTGGGAC

TGGAACTGG

**MT909568**

TGGCTTGGCTATCGCCCACCCGGATCTGGCGGACAACGTGCGTCCGGGCTCAAAAAACGTGGTGACCGGC

AGCGACGACCCGACCCCGACCGATGCGGATACGGCCCACGGCACTTCGGTCTCCGGCATCATAGCGGCGG

TAGACAACGCCATCGGCACCAAGGGGATAGCCCCGCGGGCCCAGTTGCAGGGGTTCAACCTGCTGGACGA

CAACAGCCAGCAGTTGCAGAAAGATTGGCTCTACGCCCTTGGCGACAGCGATGCCAGCCGCGACAACCGG

GTGTTCAACCAGAGTTACGCATGAGC

**MT909569**

TGGCTTGGCTATCGCCCACCCGGATCTGGCGGACAACGTGCGTCCGGGCTCAAAAAACGTGGTGACCGGC

AGCGACGACCCGACCCCGACCGATGCGGATACGGCCCACGGCACTTCGGTCTCCGGCATCATAGCGGCGG

TAGACAACGCCATCGGCACCAAGGGGATAGCCCCGCGGGCCCAGTTGCAGGGGTTCAACCTGCTGGACGA

CAACAGCCAGCAGTTGCAGAAAGATTGGCTCTACGCCCTTGGCGACAGCGATGCCAGCCGCGACAACCGG

GTGTTCAACCAGAGTTACGCATGAGC

**MT909570**

TGGCTTGGCTATCGCCCACCCGGATCTGGCGGACAACGTGCGTCCGGGCTCAAAAAACGTGGTGACCGGC

AGCGACGACCCGACCCCGACCGATGCGGATACGGCCCACGGCACTTCGGTCTCCGGCATCATAGCGGCGG

TAGACAACGCCATCGGCACCAAGGGGATAGCCCCGCGGGCCCAGTTGCAGGGGTTCAACCTGCTGGACGA

CAACAGCCAGCAGTTGCAGAAAGATTGGCTCTACGCCCTTGGCGACAGCGATGCCAGCCGCGACAACCGG

GTGTTCAACCAGAGTTACGCATGAGC

**MW001219**

GGGGTGACCCAGAAGGGGTTATCCGCCGCCGATGCGCTGCAGCTCTACTCCATCCTGACGGTGGGTGATG

GCATGGTCTCCCAGGTGCCGGCGCTGCTGATCGCCATCACCGCGGGGATCATCGTCACCCGGGTCTCCTC

CGAAGAGTCTTCCGATCTGGGTACCGATATCGGTGCCCAAGTGGTGGCCCAGCCCAAGGCGCTGCTGATA

GGTGGTCTGCTGCTGGTGCTGTTTGGTCTGATCCCGGGCTTCCCGATGATCACCTTCTTTGCGCTGGCGG

CCATCGTCACGGCCGGCGGTTATTTTATCGGCTTGCGTCAGCGCAAGGCGCAGAGCAGCAACAGTCAGGA

TCTTCCTGCCGTGCTGGCGCAGGGGGCGGGAGCCCCCGCCGCCCGCAGCAAGCCAAAACCAGGCAGCAAG

CCGCGGGGCAAGCTGGGGGAGAAGGAGGAGTTTGCCATGACGGTGCCACTCCTGATCGATGTGGATGCCG

CTTTGCAGGCCGAGCTGGAGGCCATCGCCCTCAACGATGAACTGGTACGGGTGCGCCGCGCCCTCTATCT

CGATCTCGGGGTGCCCTTCCCGGGTATTCACCTGCGTTTCAACGAGGGGA

**MW001220**

GGGGTGACCCAGAAGGGGTTATCCGCCGCCGATGCGCTGCAGCTCTACTCCATCCTGACGGTAGGTGATG

GCATGGTCTCCCAGGTGCCGGCGCTGCTGATCGCCATCACCGCGGGGATCATCGTTACCCGGGTCTCCTC

CGAAGAGTCTTCCGATCTGGGTACCGATATCGGCGCCCAGGTGGTGGCCCAGCCCAAGGCGCTGCTGATC

GGCGGTCTGTTGCTGGTGTTGTTTGGTCTGATCCCGGGCTTCCCGATGATCACCTTCTTTGCGCTGGCGG

CCATCGTCACGGCAGGCGGTTATTTTATCGGCTTGCGTCAGCGCAAGGCGCAGAGCAGCAACAGTCAGGA

TCTTCCTGCCGTGCTGGCGCAGGGGGCGGGAGCCCCCGCCGCCCGCAGCAAGCCAAAACCGGGCAGCAAG

CCGCGGGGCAAGCTGGGGGAGAAGGAGGAGTTCGCCATGACGGTGCCACTCCTGATCGATGTGGATGCCG

CTTTGCAGGCCGAGCTGGAGGCCATCGCCCTCAACGACGAACTGGTACGGGTGCGCCGCGCCCTCTATCT

CGATCTCGGGGTACCCTTCCCGGGCATTCACCTCC

**MH607887**

GGGGTGACCCAGAAGGGGTTATCCGCCGCCGATGCGCTGCAGCTCTACTCCATCCTGACGGTCGGGGATG

GCATGGTATCGCAGGTGCCGGCGCTGCTGATCGCGATCACTGCGGGGATTATCGTTACCCGGGTCTCCTC

CGAAGAATCTTCCGATCTGGGTACCGATATCGGTGCCCAAGTGGTGGCCCAGCCCAAGGCGCTGCTGATC

GGCGGTCTGCTGCTGGTGCTGTTTGGGTTGATCCCGGGCTTCCCGATGATCACCTTCTTTGCGCTGGCGG

CCATCGTCACGGCCGGCGGTTATTTTATCGGCTTGCGTCAGCGCAAGGCGCAGAGCAGCAACAGTCAGGA

TCTTCCTGCCGTGCTGGCGCAGGGGGCGGGAGCCCCCGCCGCCCGCAGCAAGCCAAAACCAGGCAGCAAG

CCGCGGGGCAAGCTGGGGGAGAAGGAGGAGTTTGCCATGACGGTGCCACTCCTGATCGATGTGGATGCCG

CTTTGCAGGCCGAGCTGGAGGCCATCGCCCTCAACGACGAACTGGTACGGGTGCGCCGCGCCCTCTATCT

CGATCTCGGGGTGCCCTTCCCGGGTATTCACCTGCGTTTCAACGAGGGGATG

**MW001221**

GGGGTGACCCAGAAGGGGTTATCCGCCGCCGATGCGCTGCAGCTCTACTCCATCCTGACGGTGGGTGATG

GCATGGTCTCCCAGGTGCCGGCGCTGCTGATCGCCATCACCGCGGGGATCATCGTGACCCGGGTCTCCTC

CGAAGAGTCTTCCGATCTGGGTACCGATATCGGTGCCCAGGTGGTGGCCCAGCCCAAGGCGCTGCTGATC

GGCGGTCTGTTGCTGGTGCTGTTTGGTCTGATCCCGGGCTTCCCGATGATCACCTTCTTTGCGCTGGCGG

CCATCGTCACGGCAGGCGGTTATTTTATCGGCTTGCGTCAGCGCAAGGCGCAGAGCAGCAACAGTCAGGA

TCTTCCTGCCGTGCTGGCGCAGGGGGCGGGAGCCCCCGCCGCCCGCAGCAAGCCAAAACCAGGCAGCAAG

CCGCGGGGCAAGCTGGGGGAGAAGGAAGAGTTTGCCATGACGGTGCCACTCCTGATCGATGTGGATGCCG

CTTTGCAGGCCGAGCTGGAGGCGATTGCCCTCAACGACGAACTGGTGCGGGTGCGCCGCGCCCTCTATCT

CGATCTCGGGGTGCCCTTCCCGGGTATTCACCTGCGTTTCAACGAGGGGATGGGTGA

**MH607890**

GGGGTGACCCAGAAGGGGTTATCCGCCGCCGATGCGCTGCAGCTCTACTCCATCCTGACGGTGGGTGATG

GCATGGTCTCCCAGGTGCCGGCGCTGCTGATCGCCATCACCGCGGGGATCATCGTCACCCGGGTCTCCTC

CGAAGAGTCTTCCGATCTGGGTACCGATATCGGTGCCCAGGTGGTGGCCCAGCCCAAGGCGCTGCTGATC

GGCGGTCTGTTGCTGGTGCTGTTTGGTCTGATCCCGGGCTTCCCGATGATCACCTTCTTTGCGCTGGCGG

CCATCGTCACGGCAGGCGGTTATTTTATCGGCTTGCGTCAGCGCAAGGCGCAGAGCAGCAACAGTCAGGA

TCTTCCAGCCGTGCTGGCGCAGGGGGCGGGAGCCCCCGCCGCCCGCAGCAAGCCAAAACCGGGCAGCAAG

CCTCGGGGCAAGCTGGGGGAGAAGGAAGAGTTCGCCATGACGGTGCCGCTCCTGATCGATGTGGATGCCG

CTTTGCAGGCCGAGCTGGAGGCGATTGCCCTCAACGACGAACTGGTGCGGGTGCGCCGCGCCCTCTATCT

CGATCTCGGGGTGCCCTTCCCGGGTATTCACCTGCGTTTCAACGAGGGGATGGGTGAAGGC

**MH607889**

GGGGTGACCCAGAAGGGGTTATCCGCCGCCGATGCGCTGCAGCTCTATTCCATACTGACGGTCGGGGATG

GCATGGTATCCCAGGTGCCGGCGCTGCTGATCGCCATCACGGCGGGGATTATCGTTACGCGGGTGTCCTC

CGAAGAGTCTTCCGATCTGGGTACCGATATCGGTGCCCAAGTGGTGGCCCAGCCCAAGGCGCTGCTGATC

GGCGGTCTGCTGCTGGTGCTGTTTGGGTTGATCCCGGGCTTCCCGATGATCACCTTCTTTGCGCTGGCGG

CCATCGTCACGGCCGGCGGTTATTTTATCGGCTTGCGTCAGCGCAAGGCGCAGAGCAGCAACAGTCAGGA

TCTTCCTGCCGTGCTGGCGCAGGGGGCGGGAGCCCCCGCCGCCCGCAGCAAGCCAAAACCAGGCAGCAAG

CCGCGGGGCAAGCTGGGGGAGAAGGAGGAGTTTGCCATGACGGTGCCACTCCTGATCGATGTGGATGCCG

CTTTGCAGGCCGAGCTGGAGGCGATCGCCCTCAACGATGAACTGGTGCGGGTGCGCCGCGCCCTCTATCT

CGATCTCGGAGTGCCCTTCCCGGGTATTCACCTGCGTTTCAACGAGGGGATGGG

**MH607888**

GGGGTGACCCAGAAGGGGTTATCCGCCGCTGATGCGCTGCAGCTCTACTCCATCCTGACGGTAGGTGATG

GCATGGTGTCCCAGGTGCCGGCGCTGCTGATCGCCATCACCGCGGGGATCATCGTTACCCGGGTCTCCTC

CGAAGAGTCTTCCGATCTGGGTACCGATATCGGTGCCCAAGTGGTGGCCCAGCCCAAGGCGCTGTTGATA

GGTGGTCTGCTGCTGGTGCTGTTTGGGTTGATCCCGGGCTTCCCGATGATCACCTTCTTTGCGCTGGCGG

CCATCGTCACGGCCGGCGGCTATTTTATCGGCTTACGTCAGCGCAAGGCGCAGAGCAGCAACAGTCAGGA

TCTTCCAGCCGTGCTGGCGCAGGGGGCAGGAGCCCCCGCCGCCCGCAGCAAGCCAAAGCCGGGCAGCAAG

CCGCGGGGCAAGCTGGGAGAGAAGGAGGAGTTCGCCATGACGGTGCCACTCTTGATTGATGTGGACGCCG

CCTTACAGGCCGAGCTGGAGGCCATTGCCTTGAACGATGAGCTGGTACGTGTGCGCCGCGCCCTATACTT

GGATCTCGGGGTACCCTTCCCGGGCATTCACCTCC

**MW001222**

GGGGTAACCCAGAAGGGGTTATCCGCCGCCGATGCGCTGCAGCTCTACTCCATCCTGACGGTGGGTGATG

GCATGGTCTCCCAGGTGCCGGCGCTGCTGATCGCCATCACCGCGGGGATCATCGTCACCCGGGTCTCCTC

CGAAGAGTCTTCCGATCTGGGTACCGATATCGGTGCCCAGGTGGTGGCCCAGCCCAAGGCGCTGCTGATC

GGCGGTCTGTTGCTGGTGCTGTTTGGTCTGATCCCGGGCTTCCCGATGATCACCTTCTTTGCGCTGGCGG

CCATCGTCACGGCAGGCGGTTATTTTATCGGCTTGCGTCAGCGCAAGGCGCAGAGCAGCAACAGTCAGGA

TCTTCCAGCCGTGCTGGCGCAGGGGGCGGGAGCCCCCGCCGCCCGCAGCAAGCCAAAACCGGGCAGCAAG

CCTCGGGGCAAGCTGGGGGAGAAGGAAGAGTTCGCCATGACGGTGCCGCTCCTGATCGATGTGGATGCCG

CTTTGCAGGCCGAGCTGGAGGCGATTGCCCTCAACGACGAACTGGTGCGGGTGCGCCGCGCCCTCTATCT

CGATCTCGGGGTGCCCTTCCCGGGTATTCACCTGCGTTTCAACGAGGGGATGGGTGAAGGC

**MT942623**

GCCAAAGATACTACATTTGGTGGTAAACAATTGCTCAACGGTGGGTATACAGGGTCTTTCCAGGTTGGTG

CAGATGCTGCTCAGACGATTACTTTTAAGATGACCTCTGCTTTTACTATCAGCGGTATTGCGGGGGCAAG

CAAAGGTAATGCCACAATTGCCACTCAGTCAACGGGCGAACCTTTCAAAATCACCGGTGTATCGAAGGTG

AAGGTTTTGTCTACCAGCATCAACAGTATCCAGGCAGCGAGTAGTGCTCAACTTGCTATGGCAAACCTTG

ACTACATGATCAAAGTGGTTGATAGCAAGCGTGCCGAATTGGGTGC

**MT942624**

ACCGCAACGCCAACGATGGTATTTCCGTTGCCCAGACCGCTGAAGGCGCCATGGACGAAGTGACCTCCAT

GCTGCAACGGATGCGTACCCTGGCTCAGCAATCCGCCAACGGCTCAAACAACACGGATGACCGTACCGCG

TTGCAGCAAGAGTACACTCAACTGATGACTGAAATTGACCGAGTGGCCAAAGACACTACCTTTGGTGGGC

AAAACCTGCTCAGTGGCGGCTACATAGGCAGTTTCCAGGTCGGTGCCGATGCCGGTCAGACCATTACGTT

CCGGATGACTTCTGCGTTTACCATCTCTGGGATGGCGTCAGCAACAAAGGGTAATGCCACTGTCACCACG

ACCACAACGGGTGAGCCGTTTACTGTTGCGAAGAGCACAAGTGGTACCGTAACTACGACAAGTATCGGTA

GTATTACGAGCGCCAAAGAGGCACAGACATCGATGGCTAATCTGGACTTTATGATTAAGGTAGTCGATAG

CAAGCGTGCGGAGCTGGGTGCGGTA

**MT942625**

AATGGTCTGGACCAAGGTAACCGTAATGCTAACGATGGTATCTCTCTGGCACAAACGGCCGAAGGGGCTA

TGGATGAAGTGACTGGCATGCTGCAGCGTATGCGGACTCTGGCACAACAGTCAGCTAACGGCTCCAACTC

CGCCAAGGATCGTGAAGCGCTGCAAAAAGAGGTGGATCAGCTTGGAGCTGAAGTAAACAGGATTTCTAAT

GCAACGACTTTTGCAGGAACCAAGCTTCTTGATGGTTCGTTTGGTGGTACTTTCCAGGTTGGTGCGGATG

CCAACCAGACTATCGGTTTCAGCTTGAGTCAAGCTGGTGGATTCAGCATCTCTGGTATTGCAGCCGCCGC

GGGTAAAGCGACAACTTTTATTAGTGGTTCAACTGCTGGTGGAATCAGCATCAGTAGCCAGAGCAATGCA

CAGGATGTGTTAGCCGCTGTCGATTCTATGTTGGAAGTGGTTGATGGCAAGCGCGCCGAGCTTGGTGCAG

TGCAA

**MT942626**

TCTTATACCCGCTTGGCATCTGGCCTGCGTATCAACAGCGCTAAAGATGATGCTGCAGGTCTTCAAATCT

CCAACCGCTTGACCTCTCAGGTCAATGGCCTAGACCAAGGTAACCGTAACGCGAACGATGGTATCTCTCT

GGCACAAACTGCCGAAGGTGCGATGGATGAAGTGACCGGCATGCTGCAGCGTATGCGTACTTTGGCACAA

CAGTCAGCGAACGGCTCCAACTCTGCCAAGGATCGTGAAGCCCTGCAAAAAGAGGTTGATCAACTGGGTG

CAGAGATCAACCGTATCTCTACTTCTACCACTTTCGCGGGGACTAAGTTGCTTGATGGTTCCTTCGGTGG

GACTTTCCAAGTGGGGGCTGATGCCAACCAGACCATCAGTTTTAGTCTATCTCAAGCCGATGGCTTCAGT

ATTTCGGGAATTTCAGCTGCCGCAGGGACATCGATCACAACTGCTACTGGGGCTACTGTGTTGGTATCAG

CTATATTTGTAGGTGGTAGTGCTGGTGGGATCAGCATAAGTAGCCAAAGTAATGCGCAGAACGTTCTTGC

TGCTGCAGATGCCATGTTAGCGATAGTGGATGGCAAACGC

**MT977537**

ATGGGTCTTTATATCAATACTAACGTTTCATCGCTTAACGCTCAGCGTAACATGGTGAATGCGACAAAAT

CACTGGATACATCCTACACCCGTCTGGCATCCGGTCTGCGCATCAACAGCGCCAAAGACGATGCTGCCGG

CCTGCAGATTTCCAACCGTTTGACCTCCCAGATCAATGGTCTGGATCAGGGCAACCGTAATGCCAATGAT

GGTATCTCTCTGGCACAAACTGCCGAAGGTGCGATGGACGAAGTGACCGGCATGTTGCAGCGCATGCGTA

CCTTGGCTCAACAATCCGCCAACGGATCCAACTCCGCCAAGGACCGCGAAGCCCTGCAAAAAGAAGTGGA

TCAGCTGGGTGCCGAGATCAACCGTATTTCCACTGCAACTACCTTTGCCGGCACCAAGCTGCTCGATGGC

TCTTTCGGTGGCACCTTCCAAGTCGGTGCGGATGCCAATCAAACTATCAGTTTTAGTCTGAGTCAAGCTG

ATGGTTTCAGTATTTCGGGAATTGCCGCAGCGGCTACTGCTACTGTCGACGTTGGTACAGTAATTGGTAC

TGCAATTGCGGTAAACACTATCTTTGTTAGTGGCAGTGCAGGC

**MT977538**

GCCAAAGATACTACATTTGGTGGTAAACAATTGCTCAACGGTGGGTATACAGGGTCTTTCCAGGTTGGTG

CAGATGCTGCTCAGACGATTACTTTTAAGATGACCTCTGCTTTTACTATCAGCGGTATTGCGGGGGCAAG

CAAAGGTAATGCCACAATTGCCACTCAGTCAACGGGCGAACCTTTCAAAATCACCGGTGTATCGAAGGTG

AAGGTTTTGTCTACCAGCATCAACAGTATCCAGGCAGCGAGTAGTGCTCAACTTGCTATGGCAAACCTTG

ACTACATGATCAAAGTGGTTGATAGCAAGCGTGCCGAATTGGGTGC

**MT977539**

ATGAGTCTGTATATCAATACCAACGTTTCATCGCTCAACGCTCAGCGTAACATGATGAACAGCACCAAAT

CCCTGGATACCTCCTACACCCGTCTGGCCTCCGGCCTGCGCATCAACAGCGCCAAGGACGATGCGGCAGG

TCTGCAGATCTCCAACCGTCTGACCTCCCAGATCAACGGTCTGGATCAGGGCAACCGCAATGCCAACGAC

GGTATCTCCCTGGCACAAACCGCGGAAGGGGCCATGGACGAAGTGACCGGCATGCTGCAGCGTATGCGAA

CCTTGGCCCAACAATCCGCCAACGGCTCCAACTCAGATTCTGATCGCGATGCGCTGCAAAAAGAGATGGA

TCAGCTGGGGGCCGAGATCAATCGTATCTCAACCGCCACTACCTTTGCAGGTACGAAGCTGCTCGATGGC

TCTTTTAGCGGCTCCTTCCAGGTGGGGGCAGATGCCAACCAGACCATTAGTTTTAACCTGAATCAGACTG

ATGGTTTCAGTATTTCCGGTATTGCGGCGGCGGCAACCAGCGTCC
